# Conformational selection of vasopressin upon V_1a_ receptor binding

**DOI:** 10.1016/j.csbj.2021.10.024

**Published:** 2021-10-18

**Authors:** Kateryna Che, Markus Muttenthaler, Dennis Kurzbach

**Affiliations:** a University Vienna, Faculty of Chemistry, Institute of Biological Chemistry, Währinger Str. 38, A-1090 Vienna, Austria; b The University of Queensland, Institute for Molecular Bioscience, 306 Carmody Rd, 4072 St Lucia, Brisbane, Queensland, Australia

**Keywords:** Molecular recognition, Conformational selection, Molecular dynamic simulations, In silico docking, NMR spectropscopy, Vasopressin, V_1a_ receptor

## Abstract

The neuropeptide vasopressin (VP) and its three G protein-coupled receptors (V_1a_R, V_1b_R and V_2_R) are of high interest in a wide array of drug discovery programs. V_1a_R is of particular importance due to its cardiovascular functions and diverse roles in the central nervous system. The structure–activity relationships underpinning ligand-receptor interactions remain however largely unclear, hindering rational drug design. This is not least due to the high structural flexibility of VP in its free as well as receptor-bound states. In this work, we developed a novel approach to reveal features of conformational selectivity upon VP-V_1a_R complex formation. We employed virtual screening strategies to probe VP’s conformational space for transiently adopted structures that favor binding to V_1a_R. To this end, we dissected the VP conformational space into three sub-ensembles, each containing distinct structural sets for VP’s three-residue C-terminal tail. We validated the computational results with experimental nuclear magnetic resonance (NMR) data and docked each sub-ensemble to V_1a_R. We observed that the conformation of VP’s three-residue tail significantly modulated the complex dissociation constants. Solvent-exposed and proline *trans*-configured VP tail conformations bound to the receptor with three-fold enhanced affinities compared to compacted or *cis*-configured conformations. The solvent-exposed and more flexible structures facilitated unique interaction patterns between VP and V_1a_R transmembrane helices 3, 4, and 6 which led to high binding energies. The presented “virtual conformational space screening” approach, integrated with NMR spectroscopy, thus enabled identification and characterization of a conformational selection-type complex formation mechanism that confers novel perspectives on targeting the VP-V_1a_R interactions at the level of the encounter complex – an aspect that opens novel research avenues for understanding the functionality of the evolutionary selected conformational properties of VP, as well as guidance for ligand design strategies to provide more potent and selective VP analogues.

## Graphical abstract

**Figure:**
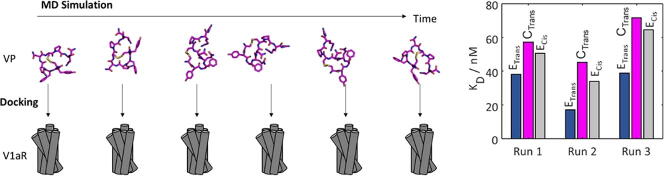

## 1 Introduction

Vasopressin (VP) is a highly conserved neuropeptide that acts *via* three class A (rhodopsin like) G protein-coupled receptors (GPCRs) [1]: vascular (V_1a_R) [2], pituitary (V_1b_R) [3], and renal (V_2_R) [4], [5]. VP regulates several vital body functions including water balance and salt homeostasis (V_2_R) [6], [7], [8], blood pressure (V_1a_R) [9], [10], pain perception (V_1a_R) [11], [12], cognitive function (V_1a_R, V_1b_R) [13], [14] and social behavior (V_1a_R, V_1b_R) [15], [16], [17], [18]. VP is also involved in the regulation of stress response (V_1b_R) [19], [20], as it takes part in the signaling mechanism associated with ACTH/cortisol release (V_1a_R) [21]. Additionally, VP plays a role in the hepatic metabolism of glucose (V_1a_R) [22], and the regulation of the sympathetic nervous system (V_1a_R, V_2_R) [23], [24]. VP is structurally similar to oxytocin (OT) and can also activate the closely related oxytocin receptor (OTR) [25]. Dysregulation of VP signaling is linked to a wide array of disorders including autism [18], [26], [27], [28], cancer development [29], [30], cardiovascular disorders [31], [32], polycystic kidney disease [33], nephrogenic diabetes insipidus [34], and nocturia [35].

VP ligand-receptor interaction has been the subject of long-standing research and drug design efforts [28], [32], [36], [37]. Nevertheless, structural dynamics and details of its receptor interactions remain unclear, particularly due to the lack of crystal or electron microscopy structures, and therapeutic targeting of its interactions has had only limited success to date [36], [37].

VP comprises a six amino acid containing, disulfide-cyclic subunit linked to an amidated three-residue C-terminal tail. Pro^7^ functions as a hinge between these two subunits [38], [39]. Arg^8^ in the three-residue tail is a primary modulator of VP-receptor (VPR) selectivity (compared to OTR) [40], [41], whose spatial position is crucial for binding [41]. Similarly, Pro^7^ plays a role in V_1a_R and V_2_R selectivity [41]. However, the structure–activity relationships (SARs) underpinning these features remain elusive.

The discrepancy between medical interest and lack of insights into structural dynamics is not least due to the limited experimental access to atomic-level details. In particular, nuclear magnetic resonance (NMR) spectroscopy – the major structure elucidation technique for non-crystallizable substrates in solution – is limited by the absence of long-range distance information (e.g., by nuclear Overhauser effects; NOE [42]). Consequently, combinations of NMR and computational techniques [38], [43] have become increasing popular to reveal the complex dynamics of VP and related SARs. In this context, we capitalized in this work on an integrative approach combining *in-silico* modelling of the VP-ligand-receptor complexes and NMR-based structure validation. More specifically, we adapted virtual screening techniques [36], [44], [45] to assess the structural dynamics involved in VPR recognition. We used the VP-V_1a_R interaction as a model system, given its particular importance for regulating cardiovascular functions.

With our approach, we demonstrate how conformational selection-type events upon encounter complex formation can be assessed. In particular, we can assess *in-silico* which structures within the heterogeneous peptide conformational space favor complex formation. Thereby, we introduce a new approach of ‘virtual conformational space screening’ at the example of a medically important target. This advancement of the virtual screening methodology enables several novel avenues for understanding peptide activities and ligand modifications in the context of SARs and drug development.

Our approach comprises two stages: First, we identified distinct sub-ensembles within the total VP structural ensemble by integrating molecular dynamics (MD) simulations and NMR spectroscopy. Then, we independently docked the identified sub-ensembles to an inactive V_1a_R-homology model [46] and screened the resulting complex structures for variations in binding energies and dissociation constants to identify preferentially selected peptide conformations.

V_1a_R preferentially selected VP conformations featuring solvent-exposed tail units, as these enabled adoption of energetically favorable complex conformations. This contrasts with two other identified sub-ensembles that feature structural restraints due to intramolecular side-chain contacts or *cis*-configuration of Pro^7^ and three-fold reduced binding energies.

Virtual screening techniques are well-established in the context of *in-silico* drug discovery [45], [47], [48] and were here expanded to peptide-receptor interactions. For virtual screening in drug-receptor interactions [44], [49], a library of drugs is typically docked to a receptor and compounds displaying the desirable binding properties are selected for further analysis. We replaced the library of drugs with a library of VP conformational ensembles to assess the conformational selectivity involved in VP-V_1a_R recognition.

Comparable approaches by Bonvin and co-workers were already successful in generating flexible protein–protein [50] and protein-peptide [51] complexes. This was achieved either by screening conformations observed in NMR structure predictions for varying binding energies [50], or by individually docking different peptides with varying degrees of conformational freedom to a target receptor [50].

Here, we first ran an MD simulation of VP in solution to obtain the library of conformations to be docked.

## 2 Results

### 2.1 Vasopressin’s conformational sub-ensembles

**Fig. 1:**
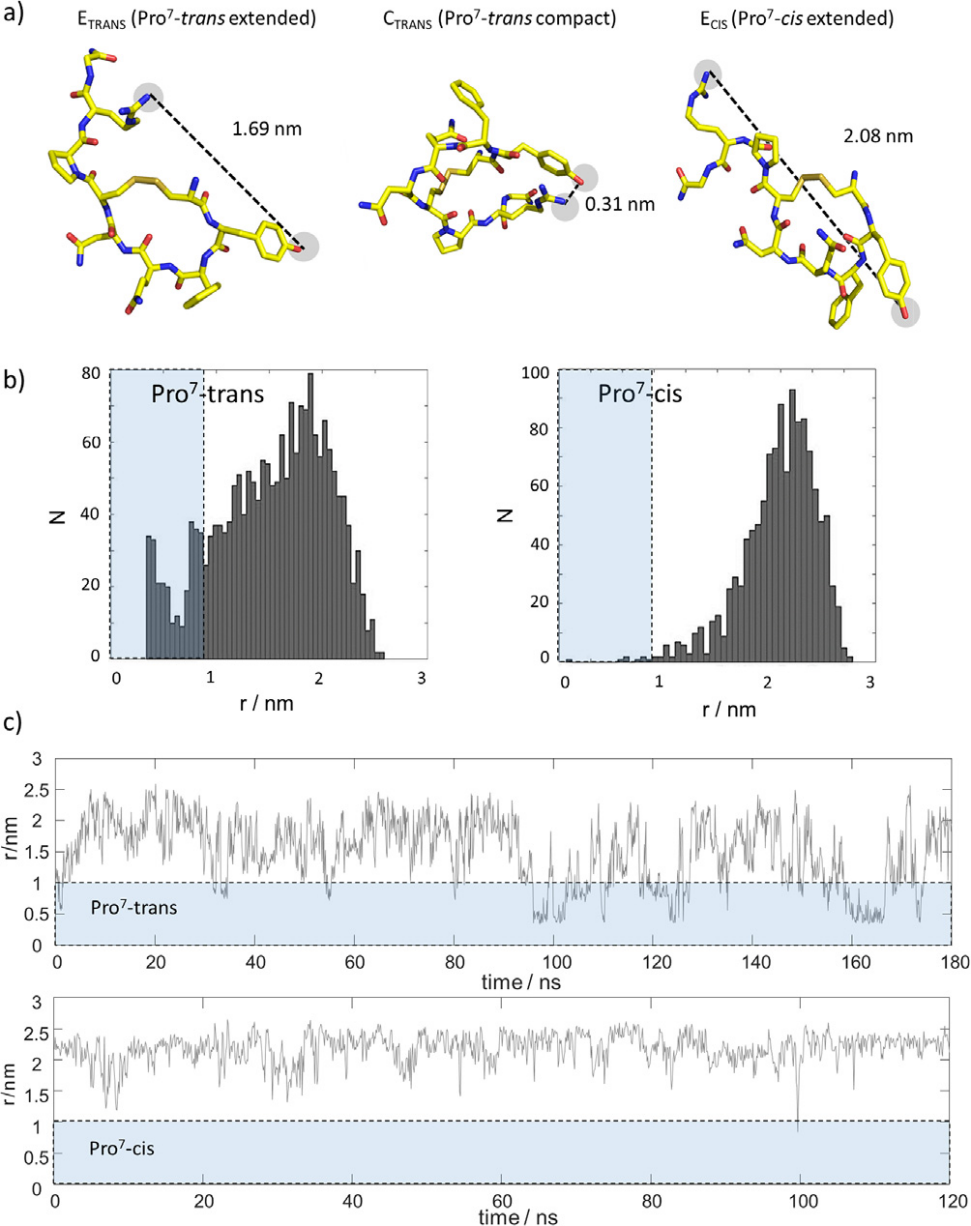
a) Representative snapshots of limiting cases for VP structures in the Pro^7^-*trans* extended (left), Pro^7^-*trans* compact (middle, most compacted) and Pro^7^-*cis* extended (right) state. The distance between residues Tyr^2^ and Arg^8^ is indicated by the dashed line. b) Distributions of Arg^8^(H^η^)-Tyr^2^(H^η^) distances *r* found in MD simulations of VP in the Pro^7^-trans (left) Pro^7^-cis (right) state. While the former displayed a bimodal distribution with two distinct maxima, the latter had only a single maximum. The blue shade indicates distances extracted from the compact sub-ensemble. c) Trajectories of Arg^8^(H^η^)-Tyr^2^(H^δ^) distances *r* underlying the distributions in panel (a). The blue shaded area guides the eye to distances < 1 nm between residues Tyr^2^ and Arg^8^. These were only observed for the Pro^7^-trans state. (For interpretation of the references to colour in this figure legend, the reader is referred to the web version of this article.)

In our MD trajectories (all-atom simulations, pH 7.4, 37 °C), VP sampled three well-distinguishable sub-ensembles including two in which Pro^7^ adopted its natural *trans* state. One of these is characterized by a compact three-residue tail and the other by an extended tail (Fig. 1a), which is in agreement with reported conformational switches [52]. The third sub-ensemble was observed when Pro^7^ adopts a *cis* state, in which the peptide tail also adopted an extended conformation. A compact sub-ensamble with Pro^7^ in a cis state was not detected (Fig. 1b). We have denoted these three observed ensembles as C_TRANS_, E_TRANS_ and E_CIS_ indicating compacted (C) and extended (E) states of the three-residue tail as well as the cis and trans conformations of Pro^7^ in the index.

In the case of the compact C_TRANS_ tail conformation, Arg^8^ folds transiently back towards Tyr^2^ and is stabilized by side-chain interactions between both residues (see Fig. 1a). In particular, the Tyr^2^-OH^η^---H^η^-Arg^8^ hydrogen bonds appear to stabilize the compacted fold. This ‘back-folded’ structure is distinct from the extended solvent-exposed tail conformations E_TRANS_ (Fig. 1a). The differential structural sampling of the two *trans* forms constitutes a bimodal conformational space, which is reflected in the intramolecular distances between atoms Arg^8^(H^η^) and Tyr^2^(H^η^). The conformational space is visualized by the distance distributions in Fig. 1b. Evidently, a bimodal distance distribution is observed. Arg^8^(H^η^)-Tyr^2^(H^η^) distances *r* as short as 0.3 nm were detected for the compacted C_TRANS_ fold. The distance distribution of this compact structural ensemble housing the back-folded state is centered at ~ 0.5 nm (Fig. 1b, left). By contrast, the extended mode that includes all other disordered states spans distances up to 2.5 nm whilst being centered at 1.9 nm (Fig. 1b, right).

When the conformation of Pro^7^ switches from a *trans* to *cis* state, only extended peptide tails were sampled (Fig. 1b, right panel, monomodal histogram). Here, the Arg^8^(H^η^)-Tyr^2^(H^η^) distances span a broad dominant distribution centered at ∼ 2.2 nm, consisting of species having separations ranging again from 1-2.8 nm. Fig. 1c displays the time trajectories underlying the distance distributions. It can be seen how VP in the Pro^7^-*trans* state switches between the two sub-ensembles, *i.e.*, compact (indicated by the blue area) and extended. No such switches were observed for the Pro^7^-*cis* state.

The three different peptide tail ensembles were confirmed in three independent MD runs (Fig. 1 shows one replica; see Supporting Information for the other two replicas; note that each run contains two independent data sets for the *trans-* and *cis*-conformations, respectively. Hence, six MD trajectories were computed in total). Note that we refrained from a quantitative assessment of the MD data due to the finite length of the trajectories. Instead, we restricted our interpretation on the observation of the three different sub-ensembles, but did not quantify their relative populations or the frequencies of conformational switches.

### 2.2 Virtual conformational space screening

**Fig. 2:**
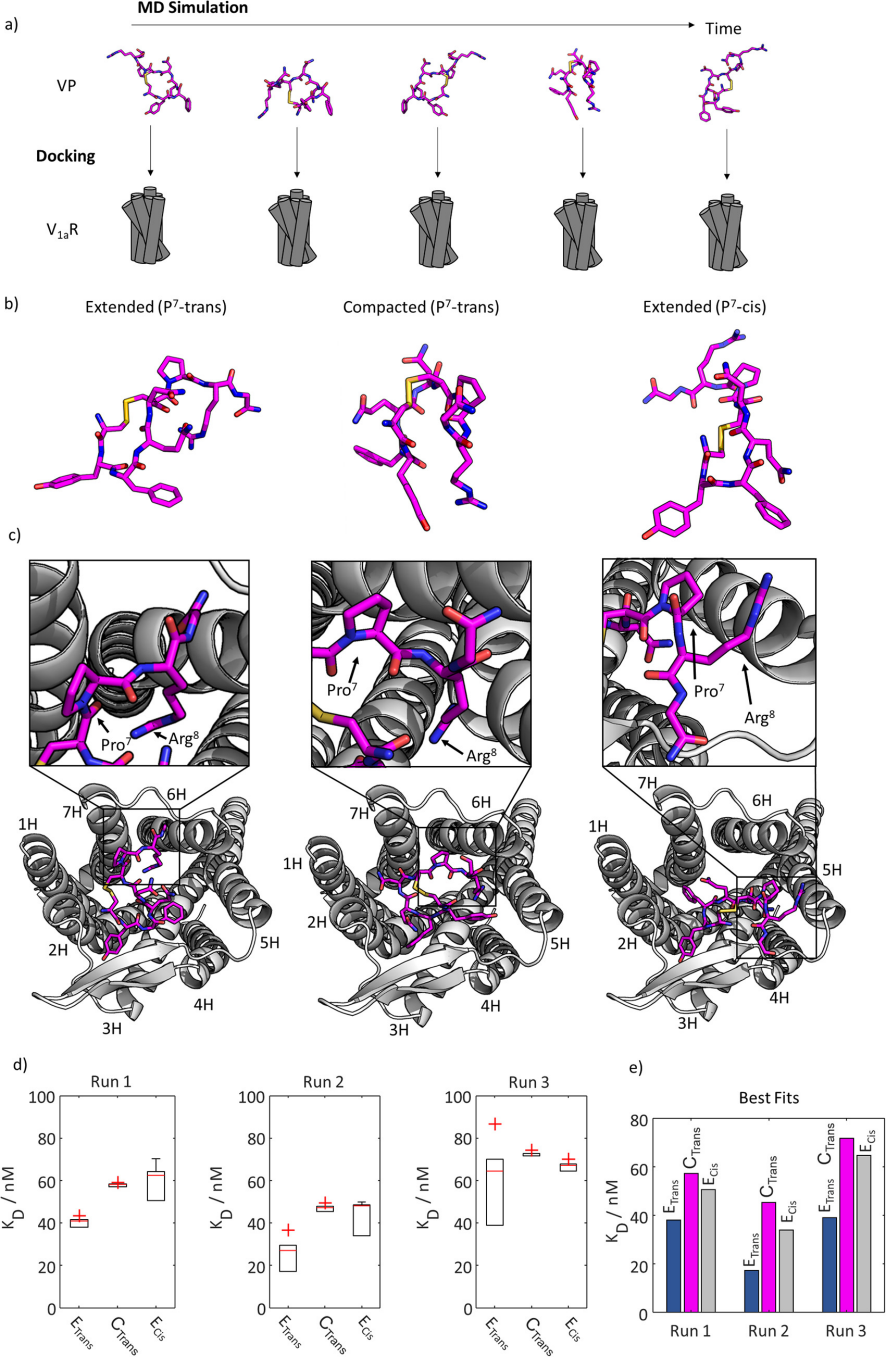
a) Sketch of the virtual conformational space screening approach. First, an MD simulations generated different sub-ensembles and conformational snapshots. Then, randomly selected structures were fed into independent docking experiments and later associated with a particular VP state. Note that the shown VP structures do not represent the conformations chosen for analysis in panels (b) to (e), but serve only as visualization of the virtual conformational screening method. b) Structures of VP in its E_TRANS_ (left), C_TRANS_ (center) and E_CIS_ (right) state. The displayed structures are VP-V_1a_R complexes with the lowest dissociation constants in our simulations. c) VP-V_1a_R complexes computed for the three structures shown in panel (b); E_TRANS_ (left), C_TRANS_ (center) and E_CIS_ (right). The transmembrane helix (TMH) numbering is indicated. Note how VP residue Arg^8^ varied its position (between TMHs 4 to 6), while Tyr^2^ and Phe^3^ always bound to TMH 3 and 4. d) Box plots of dissociation constants computed for the E_TRANS_ (left), C_TRANS_ (center) and E_CIS_ (right) sub-ensembles for three independent MD runs from the structure with the best VINA scores. The K_D_ for the E_TRANS_ sub-ensemble were always lower than those of the other two sub-ensembles. Red crosses indicate outliers, the red bars mean values, bottom and top edges of the box indicate the 25^th^ and 75^th^ percentiles, respectively. e) Representation of K_D_ for the complexes with the best scores in our docking experiments, with E_TRANS_ having significantly reduced dissociation constants. Note that these values approach the bottom edges of the boxes in panel d (see the main text for details). (For interpretation of the references to colour in this figure legend, the reader is referred to the web version of this article.)

Next, we randomly selected 50 structures from the three sub-ensembles observed in the MD trajectories and docked these *in-silico* to an established V_1a_R model [46]. For computation, we used VINA (Vina Is Not Autodock [53], [54]); further details can be found in the Experimental section. This ‘virtual conformational space screening’ approach is illustrated in Fig. 2a. From the all-atoms MD trajectories, snapshots of 50 different VP conformations were independently docked to the V_1a_R model. The VP structures for each of the three sub-ensembles (C_TRANS_, E_TRANS_ and E_CIS_) that led to the lowest dissociation constants are depicted in Fig. 2b and the associated ligand-receptor complex structures in Fig. 2c.

It was interesting to observe how residues Tyr^2^ and Phe^3^ interacted with transmembrane helices (TMH) 3 and 4 in all cases to provide optimal complex stability (in agreement with published results [41]). In contrast, the position of the three-residue tail varied for the three docked cases C_TRANS_, E_TRANS_ and E_CIS_. The preformed tail structures were conserved upon docking to the receptor which led to different complex conformations. Most prominently, tail residue Arg^8^ interacted with TMH 6 in the E_TRANS_, but with TMH 5 for the C_TRANS_ and E_CIS_ states, while tail residue Gly^9^ interacted with TMH 6 in the E_TRANS_ and C_TRANS_ states, but with TMH 4 in the E_CIS_ state. These findings are in partial agreement with published structures of the VP-V_1a_R complex. These displayed similar interaction patterns for the three-residue tail with TMH 4 [41], [55]. Yet, the interaction with TMH 6 was not reported.

For statistical analysis, each docking experiment for the 50 selected VP conformations was repeated 25 times yielding 1250 VP-V_1a_R complexes per sub-ensemble. Each computed complex structure was analyzed with respect to binding energies and dissociation constants *K_D_*. Fig. 2d displays box plots for the three sub-ensembles C_TRANS_, E_TRANS_ and E_CIS_, calculated in each case from the top five ligand-receptor complex structures with respect to binding energies. The same analysis was performed for three independent runs. In each run, the E_TRANS_ state led to significantly lower dissociation constants compared to C_TRANS_ and E_CIS_. The p-values for comparing the compacted and extended conformations the three runs were 1.0⋅10^−5^, 1.6⋅10^−3^ and 1.9⋅10^−1^. The full list of computed values is in the Supporting Information (Table S1). Our findings were further confirmed by comparison of the dissociation constants calculated for the complex structure with the best VINA score out of the 1250 structures (Fig. 2e). E_TRANS_ led in all runs to two- to three-fold reduced dissociation constants. Note that several structures with comparably low dissociation constants have been computed (see Table S1). Since only results with the five highest VINA scores were used for computation of the box plots in Fig. 2d, the bottom edges converge to the values shown in Fig. 2e.

### 2.3 NMR validation of the VP structure: The heterogeneous conformational space of the VP Pro^7^-trans form

**Fig. 3:**
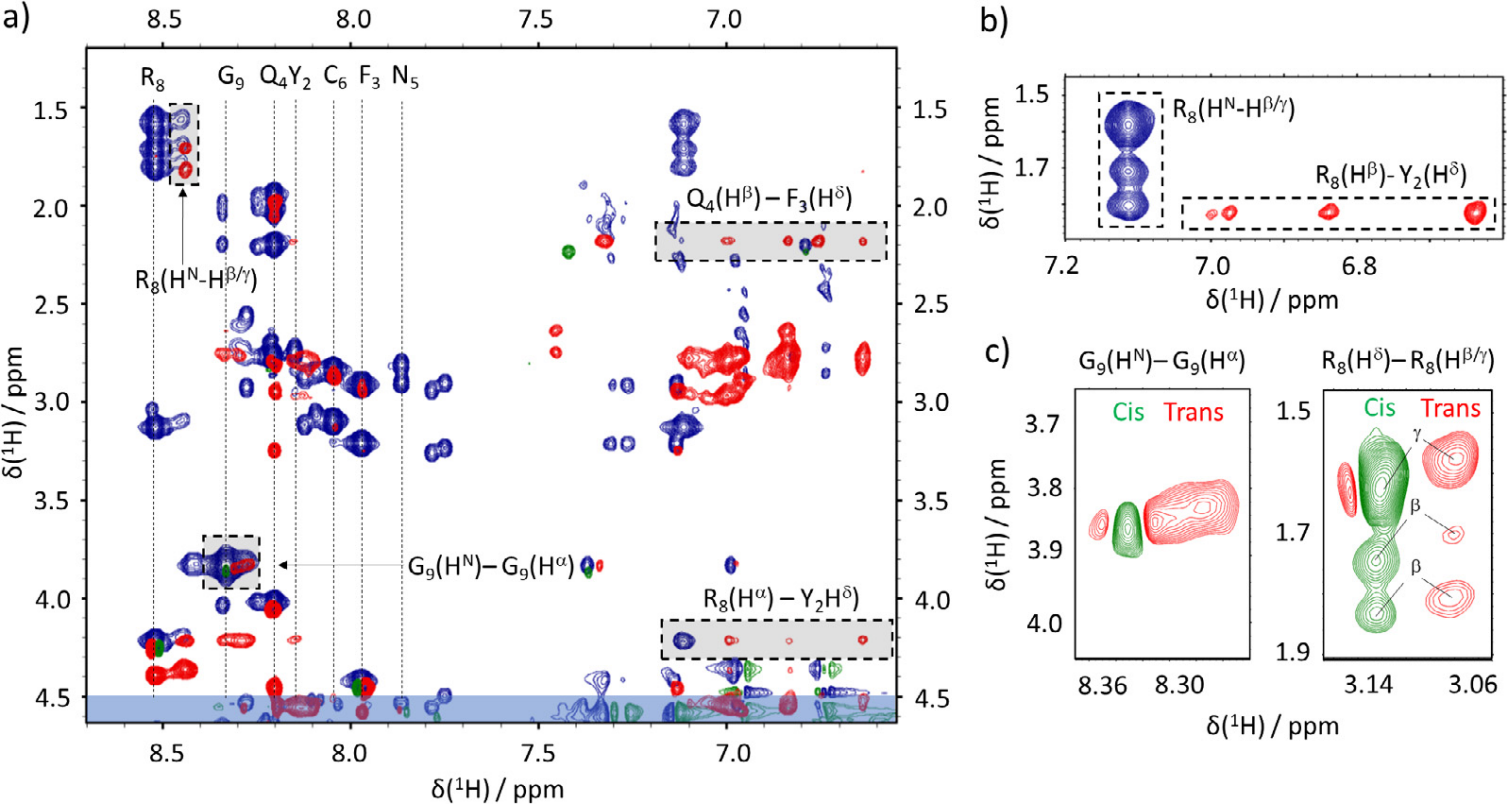
Overlay of an ^1^H–^1^H TOCSY (blue) and ^1^H–^1^H NOESY (red – positive values and green – negative values) of VP obtained at a proton Larmor frequency of 600 MHz. Spectra were recorded at 25° C and pH 6.5. a) NOESY correlations of residues of interest are marked with dashed rectangles. Cross-peaks with the water signal are marked with a blue semi-transparent rectangle. For 1D projections see the Supporting Information. b) NOESY correlations (red) between Y^2^(H^δ^) and R^8^(H^β^) are marked together with TOCSY correlations (blue) between R^8^(H^β/γ^) and R^8^(H^N^). c) Zoom onto NOESY spectra showing signals for amino acids G^9^ and R^8^. (For 1D projections see the Supporting Information.) The cross-peak amplitudes feature inverted signs for the *trans* and *cis* conformation, as only the *trans* form entails that compacted conformational ensemble, which slows down side-chain dynamics and leads to positive NOEs with respect to a positively phased diagonal. The Greek letters indicate the R^8^ protons that couple to the H^δ^. (For interpretation of the references to colour in this figure legend, the reader is referred to the web version of this article.)

To validate the MD-based results, we experimentally probed VP’s conformational space by means of solution-state NMR spectroscopy. Evidence for the tri-modal sampling space of the three-residue tail was observed in ^1^H–^1^H NOESY and ^1^H–^1^H TOCSY experiments. An overlay of TOCSY (blue) and NOESY (red-green) NMR spectra of VP in phosphate buffered solution at pH 6.5 is presented in Fig. 3a. The resonances of Gly^9^ and Arg^8^ are both split into two clear signals along the direct dimension, indicating the presence of the *cis* and *trans* Pro^7^ isoforms (further confirmation by repeated annealing is presented in the Supporting Information). Additionally, three features are notable in the NOESY data: (*i*) Arg^8^(H^α^)-Gly^9^(H^α^) cross-peaks were observed for the Pro^7^-*trans* state, but not for the *cis* state, (*ii*) Arg^8^(H^α^)-Tyr^2^(H^δ^) cross-peaks were observed (Fig. 3b; note that these NOEs contacts stem from different atoms than the H-bond described in Fig. 1; as the Arg^8^(H^η^) could not be identified by NMR.), and, most importantly, (*iii*) the Arg^8^(H^β^)-Arg^8^(H^N^) and the Arg^8^(H^γ^)- Arg^8^(H^N^) cross-peaks had a negative amplitude for the Pro^7^-*cis* and a positive one for the Pro^7^-*trans* state (with respect to positively phased diagonal peaks; green signals represent fast motions and red restricted motions; Fig. 3c).

The presence of these features confirmed the conformational sampling of the MD simulations for the acyclic Pro^7^-Arg^8^-Gly^9^ tail. In particular, the positive amplitudes for the Arg^8^(H^β^)-Arg^8^(H^N^) and the Arg^8^(H^γ^)-Arg^8^(H^N^) NOE cross-peaks for the Pro^7^-*trans* state indicate slowed conformational dynamics of the three-residue tail. By contrast, in the Pro^7^-*cis* state, only negative cross-peaks were observed indicating faster side-chain dynamics. This validated the heterogeneous conformational space of the VP three-residue tail, as only transient intramolecular attraction between the tail and the macrocycle residues could account for these observations [52]. Given the observation of Arg^8^(H^β^)-Tyr^2^(H^δ^) cross-peaks, attractive interaction between tail residue Arg^8^ and the Tyr^2^ aromatic side-chain appear intuitive as an underlying force, which aligned well with the structures computed for the C_TRANS_ case.

We conclude that VP, when Pro^7^ adopts the *trans* state, indeed populates a bimodal conformational space due to transient side-chain interactions between Tyr^2^ and Arg^8^ that slows down conformational sampling, *i.e.*, the intramolecular dynamics. In the Pro^7^-*cis* state, no indications of a conformationally restrained sub-ensemble were observed, and hence no compacted state could be inferred, in line with the MD results. Besides, the observation of slow conformational dynamics for the *trans*-state indicates that the C_TRANS_ forms dominate the conformational ensemble when Pro^7^ adopts the *trans*-state. Indeed, a predominance of extended conformations likely would result in fast backbone and side-chain dynamics and a similar cross-peak sign as observed for the E_CIS_ structural ensemble.

Note that a line shape analysis supports this conclusion. The *trans*-state leads to a broader linewidth compared to the *cis*-state (Fig. 3c and S5-S7 in the Supporting Information, the *trans*-state G^9^ peak even overlaps with the *cis*-state one). This indicates, in agreement with the above interpretation, an exchange-based line broadening as the *trans*-state exchanges between two conformations. However, the *cis*-state only samples extended forms such that no exchange broadening is observed.

It has been suggested that both the *cis* and *trans* conformations of VP are populated under physiological conditions, owing to the similarity in energy of their respective proline backbone bond configurations [39], [56], [57]. At the same time, the energy barrier for bond rotation is sufficiently high to permit observation of the separate resonances for both isomers on the NMR time scale [58]. Indeed, previous NMR investigations of VP structure observed spectroscopic fingerprints of two isomers, observing Pro^7^-*cis* populations in the range of 5–9% [56], [38] under near-physiological conditions. Here, under our conditions (pH 6.5, 25 °C), we were able to detect the presence both states simultaneously. To this end, we used the almost pure *cis*-form as starting material and obtained ~ 60% *trans* populations (as judged from the NMR peak intensities) after annealing at 60 °C for 8 h.

## 3 Discussion

V_1a_R is the main receptor in mediating these functions [59] and it is thus not surprising that significant efforts have been made to develop selective V_1a_R agonists and antagonists for therapeutic applications as well as to study this signaling system [60], [61].

Targeting V_1a_R has been a long-standing research challenge [28], [37], [46], [62], [63] mainly due to the selectivity problems to its closely-related receptor subtypes, namely V_1b_R, V_2_R and OTR. Our findings might therefore be of interest to the medicinal chemistry community for the development of more potent and selective V_1a_R ligands. In particular, one could envisage to implement unnatural amino acids that shift the C_TRANS_/E_TRANS_ population equilibrium to improve receptor affinity and potentially also receptor subtype selectivity. Our simulations indicate that the Tyr^2^-OH--–NH-Arg^8^ hydrogen bond particularly stabilizes the C_TRANS_ state. Hence, modification of either the Tyr^2^ or the Arg^8^ residue might foster an overpopulation of the better binding E_TRANS_ state, e.g., by introducing a methylated Tyr^2^ modification that lacks the possibility to form the hydrogen bond in question. Alternatively, the binding affinity of VP to V_1a_R could be modulated by directly manipulating the *cis*–*trans* equilibrium of the Pro^7^ residue [64], [65]. Such strategic modifications could deliver important potency and selectivity improvements for V_1a_R binding, which is critical for therapeutic lead development.

Concerning the computed complex structures, we found that the dissociation constant of the VP-V_1a_R complex depends on the spatial conformation of the VP three-residue tail. The lower dissociation constant for E_TRANS_ indicates a preferential binding of the corresponding structural sub-ensemble to the receptor. From Fig. 2c we deduce that the conformation with an extended peptide tail enables simultaneous interactions of VP residue Arg^8^ with TMH 6, accompanied simultaneously by interactions aromatic sidechains of Tyr^2^ and Phe^3^ with TMHs 2 and 4, respectively. This complex configuration led to the highest binding energies in our simulations but was not observed in the E_CIS_ nor C_TRANS_ states, suggesting that this unique configuration drives the observed conformational selectivity. This is supported by other studies stating that the VP ligand-receptor complex is characterized by an intricate network of hydrogen bond interactions across several residues, rather than by a few well-defined points of contact [66]. The ligand-receptor interactions in the complex consist primarily of contacts between the residues of the cyclic part of VP and TMHs 3 and 4 and the extracellular loops of the receptor [55], [66]. At the same time, the C-terminal tail modulates binding affinities *via* a few intermolecular contacts [66]. Indeed, SAR studies of VP revealed that modification or replacement of Pro^7^ in VP can affect V_1a_/V_2_ receptor selectivity [67], [68].

It should be noted that the herein computed differences in dissociation constants between the compacted and extended VP conformations likely do not suffice to lead to a ‘pure’ conformational selection-type binding event. Instead, a more complex binding process that features aspects of structural selectivity appears to be more probable. Moreover, due to the nature of virtual docking experiments with limited receptor flexibility, the computed dissociation constants correspond to conformations found upon ligand-receptor encounter. In other words, the E_TRANS_ state is preferentially selected by V_1a_R for encounter complex formation as the likelihood of dissociation is significantly reduced in comparison to the C_TRANS_ and E_CIS_ VP states_._ However, subsequent conformational sampling and structural adaption in the complex are not excluded by the presented methodology. In contrast, it is not unlikely that the selectivity towards a particular encounter complex structure can guide the VP-V_1a_R system towards a particular final complex structure.

VP has received substantial attention from both, the structural biology [36], [41], [55] and medicinal chemistry communities [28], [69], [70], and V_1a_R remains a drug target of interest. However, crystallization and electron microscopy efforts so far have failed to provide a high-resolution structure of this receptor. With longer MD trajectories, higher computing power, more refined V_1a_R homology models, and high-sensitivity NMR becoming more accessible, deeper insights into the conformational characteristics of VP and its receptor interactions can be provided. We capitalized on these developments in this study and adapted a well-established *in-silico* methodology for screening receptor interactions of potential drug candidates to the screening of solution conformations for favorable binding properties. In classical virtual screening experiments, the rational selection of the ligand set often constitutes a bottleneck, but we could overcome this by confirming the selected conformations experimentally by NMR spectroscopy.

## 4 Conclusions

We developed a ‘virtual conformational space screening’ that provided new insights into the interactions of VP with V_1a_R. We could establish that the binding event has different energetic preferences based on the sampled and experimentally verified conformations of the VP three-residue tail. In particular, the extended conformations of the Pro^7^-*trans* configuration (E_TRANS_) led to a reduced dissociation constant and favorable binding energies.

Our results highlight the key role of the three-residue ‘tail’ in determining VP conformational selectivity, providing missing atomic-level detail to the current understanding of VP-V_1a_R interaction – an aspect that opens novel research avenues for understanding the functionality of the evolutionary selected conformational properties of VP, as well as guidance for ligand design strategies to provide more potent and selective VP analogues. Our VCSS is fast, easy to implement, applicable to other peptide-receptor systems, and data interpretation is straightforward. We expect this methodological advance to be interesting for a spectrum of chemists and biologists as it enables the elucidation of peptide-receptor recognition events at the atomic level.

## 5 Experimental

VP was obtained from Biosynth Carbosynth (Compton, United Kingdom).

### 5.1 NMR spectroscopy

For NMR spectroscopy VP was dissolved at 3 mg/mL in PBS buffer at pH 6.0 (90% H_2_O and 10% D_2_O; 25 mM Na_2_HPO_4_, 25 mM KH_2_PO_4_, 25 mM NaCl). NMR spectra were recorded on a 600 MHz Bruker NEO spectrometer equipped with a Prodigy TCI probe head. All spectra were acquired at 25 °C. TOCSY and NOESY data were recorded using the Bruker ‘dipsi2gpph19′ and ‘noesyfpgpphrs19′ pulse sequences for TopSpin 4. For annealing, the samples were heated to a maximum of 60 °C for 8 h. The high temperatures were necessary as the cis–trans conversion features high energy barriers and is rather slow. All TOCSY spectra were recorded with a spectral width of 8196.7 Hz in both dimensions and 32 scans. Mixing time was 150 ms. QUADRATURE detection was done using States-TPPI. All NOESY spectra were measured with width of 9615.3 Hz in the F2 dimension and 7202.1 Hz in the F1 dimension. We recorded 32 scans. The mixing time was 300 ms. QUADRATURE detection was employed again using States-TPPI sampling schemes.

Spectral processing was achieved using TopSpin, NMRPipe [71], and Sparky [72]. All data were zero filled to twice the original number of data points and apodized using a 60° shifted sine bell function prior to Fourier transformation. This was followed by a polynomial baseline correction in the frequency space.

Line shape analysis were conducted using home-written scripts for the MATLAB software package employing the ‘fitnlorentian.m’ function.

### 5.2 MD simulations

We performed all-atoms MD simulations of VP, beginning from either Pro^7^-*cis*- or -*trans* conformations. The published Pro^7^-*trans* VP structure was used as starting model [73]. To obtain the Pro^7^-*cis* form the N-C’ bond was switched accordingly. This was followed in both cases by energy minimization and simulated annealing in explicit solvent (1% NaCl in water at pH 7.4). Finally, trajectories were recorded at 37 ˚C at 2 fs time steps.

MD-simulations were performed using the YASARA software-package [74], [75]. The AMBER03 force field was employed with periodic boundary conditions [76]. Non-bonded interactions were cut off at 1.05 nm Long-range Coulombic interactions were treated by a smoothed particle-mesh Ewald method [77], [78]. Non-canonic amino acids were built using YASARA and semi-quantum-mechanically parameterized (YAPAC-AM1). MD trajectories of > 120 ns length were accumulated for the two systems. In total, six trajectories were computed, *i.e.*, for both states (*cis* and *trans*) three trajectories were computed. Intermolecular forces were recalculated at every second simulation sub-step. Temperature rescaling was employed with a set-temperature of 37 °C. The box dimensions (cubic of 37 Å side length) were controlled to yield a solvent pressure of 1 bar. Snapshots of the simulations were taken every 10,000 fs.

### 5.3 Receptor docking

Insights into ligand-receptor interactions were generated using VINA [53] as implemented in YASARA. We used a recently reported homology model of V_1a_R [46]. 25 structures were generated for each docked structure. Side-chains flexibility was enabled upon docking the ligand structures. Dissociation constants were calculated from the binding energies ΔG_binding_ following the Autodock method and conventions:

$$K_{\text{Binding}}=1/K_{D}$$

$$\text{ln}K_{\text{Binding}}=-\text{ln}K_{D}$$

$$\Delta G_{\text{binding}}=-RT\;\text{ln}K_{\text{Binding}}$$

The binding energy ΔG_binding_ was calculated using the ‘BindEnergy’ macro implemented in YASARA, *i.e.*, by determining the energy at infinite distance (between the object and the rest of the soup, *i.e.*, the unbound state) and then subtracting the energy of the soup (the bound state). The more positive the binding energy, the more favorable the interaction. It should be noted that such determined energies are representing approximations that should only be compared among each other. They should not be understood as independent absolute measures of binding affinities. For each of the MD runs, the three sub-ensembles were identified, and the respective docking experiments were performed. This led to a total of nine docking experiments. The initial VP structure for docking to V_1a_R were chosen randomly from the respective conformational sub-ensembles using the MATLAB random number generator. Box plots (Fig. 2) were calculated using the ‘boxplot’ function implemented in the MATLAB software package.

#### CRediT authorship contribution statement

**Kateryna Che:** Investigation, Methodology, Visualization. **Markus Muttenthaler:** Conceptualization, Funding Aquisitoion, Writing – review & editing. **Dennis Kurzbach:** Conceptualization, Funding Aquisitoion, Writing – original draft.

## Declaration of Competing Interest

The authors declare that they have no known competing financial interests or personal relationships that could have appeared to influence the work reported in this paper.

## References

[1] Thibonnier M. (1998). Molecular pharmacology of human vasopressin receptors. Adv Exp Med Biol.

[2] Morel A., O'Carroll A.-M., Brownstein M.J., Lolaft S.J. (1992). Molecular cloning and expression of a rat Via arginine vasopressin receptor. Nature.

[3] Sugimoto T. (1994). Molecular cloning and functional expression of a cDNA encoding the human V1b vasopressin receptor. J Biol Chem.

[4] Birnbaumer M., Seibold A., Gilbert S., Ishido M., Barberis C., Antaramian A. (1992). Molecular cloning of the receptor for human antidiuretic hormone. Nature.

[5] Lolait S.J., O'Carroll A.-M., McBride O.W., Konig M., Morel A., Brownstein M.J. (1992). Cloning and characterization of a vasopressin V2 receptor and possible link to nephrogenic diabetes insipidus. Nature.

[6] Robertson G.L., Shelton R.L., Athar S. (1976). The osmoregulation of vasopressin. Kidney Int.

[7] Boone M., Deen P.M.T. (2008). Physiology and pathophysiology of the vasopressin-regulated renal water reabsorption. Pflugers Arch.

[8] Bankir L., Bouby N., Ritz E. (2013). Vasopressin: a novel target for the prevention and retardation of kidney disease?. Nat Rev Nephrol.

[9] Cowley A.W., Monos E., Guyton A.C. (1974). Interaction of vasopressin and the baroreceptor reflex system in the regulation of arterial blood pressure in the dog. Circ Res.

[10] Smith P.M., Ferguson A.V. (1997). Vasopressin acts in the subfornical organ to decrease blood pressure. Neuroendocrinology.

[11] Thurston C.L., Culhane E.S., Suberg S.N., Carstens E., Watkins L.R. (1988). Antinociception vs motor effects of intrathecal vasopressin as measured by four pain tests. Brain Res.

[12] Kordower J.H., Bodnar R.J. (1984). Vasopressin analgesia: specificity of action and non-opioid effects. Peptides.

[13] Weingartner H., Gold P., Ballenger J.C., Smallberg S.A., Summers R., Rubinow D.R. (1981). Effects of vasopressin on human memory functions. Science.

[14] Abramova O., Zorkina Y., Ushakova V., Zubkov E., Morozova A., Chekhonin V. (2020). The role of oxytocin and vasopressin dysfunction in cognitive impairment and mental disorders. Neuropeptides.

[15] Heinrichs M., von Dawans B., Domes G. (2009). Oxytocin, vasopressin, and human social behavior. Front Neuroendocrinol.

[16] Albers H.E. (2012). The regulation of social recognition, social communication and aggression: Vasopressin in the social behavior neural network. Horm Behav.

[17] Insel T.R., O’Brien D.J., Leckman J.F. (1999). Oxytocin, vasopressin, and autism: is there a connection?. Biol Psychiatry.

[18] Dumais K.M., Veenema A.H. (2016). Vasopressin and oxytocin receptor systems in the brain: Sex differences and sex-specific regulation of social behavior. Front Neuroendocrinol.

[19] Gibbs D.M. (1986). Vasopressin and oxytocin: hypothalamic modulators of the stress response: a review. Psychoneuroendocrinology.

[20] Stevenson E.L., Caldwell H.K. (2012). The vasopressin 1b receptor and the neural regulation of social behavior. Horm Behav.

[21] Senn M., Maier P.M., Langhans W. (1995). ACTH, cortisol and glucose responses after administration of vasopressin in cattle and sheep. J Comp Physiol B.

[22] Hems D.A., Whitton P.D. (1973). Stimulation by vasopressin of glycogen breakdown and gluconeogenesis in the perfused rat liver. Biochem J.

[23] Kvetňanský R., Porter J.C., Ježová D. (1990). Circulating regulatory factors and neuroendocrine function.

[24] Hasser E.M. (2000). Area postrema and sympathetic nervous system effects of vasopressin and angiotensin II. Clin Exp Pharmacol Physiol.

[25] Kimura T., Tanizawa O., Mori K., Brownstein M.J., Okayama H. (1992). Structure and expression of a human oxytocin receptor. Nature.

[26] Oztan O., Garner J.P., Constantino J.N., Parker K.J. (2020). Neonatal CSF vasopressin concentration predicts later medical record diagnoses of autism spectrum disorder. Proc Natl Acad Sci.

[27] Carson D.S., Garner J.P., Hyde S.A., Libove R.A., Berquist S.W., Hornbeak K.B. (2015). Arginine vasopressin is a blood-based biomarker of social functioning in children with autism. PLoS ONE.

[28] Schnider P. (2020). Discovery of balovaptan, a vasopressin 1a receptor antagonist for the treatment of autism spectrum disorder. J Med Chem.

[29] Péqueux C. (2004). Oxytocin- and vasopressin-induced growth of human small-cell lung cancer is mediated by the mitogen-activated protein kinase pathway. Endocr Relat Cancer.

[30] Taylor A.H. (1990). Interaction of vasopressin and oxytocin with human breast carcinoma cells. Cancer Res.

[31] Goldsmith S.R., Francis G.S., Cowley A.W., Goldenberg I.F., Cohn J.N. (1986). Hemodynamic effects of infused arginine vasopressin in congestive heart failure. J Am Coll Cardiol.

[32] Mondritzki T. (2021). Cardiac output improvement by pecavaptan: a novel dual-acting vasopressin V1a/V2 receptor antagonist in experimental heart failure. Eur J Heart Fail.

[33] Wang X., Wu Y., Ward C.J., Harris P.C., Torres V.E. (2008). Vasopressin directly regulates cyst growth in polycystic kidney disease. J Am Soc Nephrol.

[34] Oksche A., Rosenthal W. (1998). The molecular basis of nephrogenic diabetes insipidus. J Mol Med.

[35] Natsume O. (2006). A Clinical investigation of nocturnal polyuria in patients with nocturia: a diurnal variation in arginine vasopressin secretion and its relevance to mean blood pressure. J Urol.

[36] Hagiwara Y. (2013). Molecular modeling of vasopressin receptor and *in silico* screening of V1b receptor antagonists. Expert Opin Drug Discov.

[37] Ali F., Guglin M., Vaitkevicius P., Ghali J.K. (2007). Therapeutic potential of vasopressin receptor antagonists. Drugs.

[38] Sikorska E., Rodziewicz-Motowidło S. (2008). Conformational studies of vasopressin and mesotocin using NMR spectroscopy and molecular modelling methods. Part I: studies in water. J Pept Sci.

[39] Yedvabny E., Nerenberg P.S., So C., Head-Gordon T. (2015). Disordered structural ensembles of vasopressin and oxytocin and their mutants. J Phys Chem B.

[40] Saleh N. (2016). A Three-site mechanism for agonist/antagonist selective binding to vasopressin receptors. Angew Chem Int Ed.

[41] Ślusarz M.J., Giełdoń A., Ślusarz R., Ciarkowski J. (2006). Analysis of interactions responsible for vasopressin binding to human neurohypophyseal hormone receptors—molecular dynamics study of the activated receptor–vasopressin–Gα systems. J Pept Sci.

[42] Schirmer R.E., Noggle J.H., Davis J.P., Hart P.A. (1970). Determination of molecular geometry by quantitative application of the nuclear Overhauser effect. J Am Chem Soc.

[43] Haensele E. (2016). Can simulations and modeling decipher NMR data for conformational equilibria? Arginine-vasopressin. J Chem Inf Model.

[44] McInnes C. (2007). Virtual screening strategies in drug discovery. Curr Opin Chem Biol.

[45] Doupnik C.A., Parra K.C., Guida W.C. (2015). A computational design approach for virtual screening of peptide interactions across K + channel families. Comput Struct Biotechnol J.

[46] Di Giglio M.G. (2017). Development of a human vasopressin V 1a -receptor antagonist from an evolutionary-related insect neuropeptide. Sci Rep.

[47] Leffler A.E. (2017). Discovery of peptide ligands through docking and virtual screening at nicotinic acetylcholine receptor homology models. Proc Natl Acad Sci.

[48] Damjanovic J., Miao J., Huang H., Lin Y.-S. (2021). Elucidating solution structures of cyclic peptides using molecular dynamics simulations. Chem Rev.

[49] Forli S. (2016). Computational protein–ligand docking and virtual drug screening with the AutoDock suite. Nat Protoc.

[50] Kurkcuoglu Z., Bonvin A.M.J.J. (2020). Pre- and post-docking sampling of conformational changes using ClustENM and HADDOCK for protein-protein and protein-DNA systems. Proteins Struct Funct Bioinforma.

[51] Trellet M., Melquiond A.S.J., Bonvin A.M.J.J., Keskin O. (2013). A unified conformational selection and induced fit approach to protein-peptide docking. PLoS ONE.

[52] Haensele E., Banting L., Whitley D.C., Clark T. (2014). Conformation and dynamics of 8-Arg-vasopressin in solution. J Mol Model.

[53] Trott O., Olson A.J. (2010). AutoDock Vina: improving the speed and accuracy of docking with a new scoring function, efficient optimization and multithreading. J Comput Chem.

[54] Jaghoori M.M., Bleijlevens B., Olabarriaga S.D. (2016). 1001 Ways to run AutoDock Vina for virtual screening. J Comput Aided Mol Des.

[55] Mouillac B. (1995). The binding site of neuropeptide vasopressin V1a receptor: evidence for a major localization within transmembrane regions. J Biol Chem.

[56] Larive C.K., Guerra L., Rabenstein D.L. (1992). Cis/trans conformational equilibrium across the cysteine6-proline peptide bond of oxytocin, arginine vasopressin, and lysine vasopressin. J Am Chem Soc.

[57] Larive C.K., Rabenstein D.L. (1993). Dynamics of cis/trans isomerization of the cysteine6-proline peptide bonds of oxytocin and arginine-vasopressin in aqueous and methanol solutions. J Am Chem Soc.

[58] Dyson H.J., Rance M., Houghten R.A., Lerner R.A., Wright P.E. (1988). Folding of immunogenic peptide fragments of proteins in water solution: I. Sequence requirements for the formation of a reverse turn. J Mol Biol.

[59] Koshimizu T. (2012). Vasopressin V1a and V1b receptors: from molecules to physiological systems. Physiol Rev.

[60] Manning M, et al. Peptide and non-peptide agonists and antagonists for the vasopressin and oxytocin V1a, V1b, V2 and OT receptors: research tools and potential therapeutic agents. In: Progress in brain research, Elsevier, 2008, vol. 170, p. 473–512. 10.1016/S0079-6123(08)00437-8 18655903

[61] Manning M. (2012). Oxytocin and Vasopressin Agonists and Antagonists as Research Tools and Potential Therapeutics. J Neuroendocrinol.

[62] Park S.E. (2020). Luteolin, a Potent Human Monoamine Oxidase-A Inhibitor and Dopamine D _4_ and Vasopressin V _1A_ Receptor Antagonist. J Agric Food Chem.

[63] Andrés M., Trueba M., Guillon G. (2002). Pharmacological characterization of F-180: a selective human V1a vasopressin receptor agonist of high affinity. Br J Pharmacol.

[64] Mateos B. (2020). The ambivalent role of proline residues in an intrinsically disordered protein: from disorder promoters to compaction facilitators. J Mol Biol.

[65] Pal D. (1999). Cis peptide bonds in proteins: residues involved, their conformations, interactions and locations. J Mol Biol.

[66] Barberis C., Mouillac B., Durroux T. (1998). Structural bases of vasopressin/oxytocin receptor function. J Endocrinol.

[67] Botos C.R., Smith C.W., Chan Y.-L., Walter R. (1979). Synthesis and biological activities of arginine-vasopressin analogs designed from a conformation-activity approach. J Med Chem.

[68] Rodziewicz-Motowidło S. (2002). Conformational solution studies of [Sar7]desamino- and [MeAla7]desamino- vasopressin analogues using NMR spectroscopy. J Pept Sci.

[69] Dekan Z. (2021). Nature-inspired dimerization as a strategy to modulate neuropeptide pharmacology exemplified with vasopressin and oxytocin. Chem Sci.

[70] Bolognani F., del Valle Rubido M., Squassante L., Wandel C., Derks M., Murtagh L. (2019). A phase 2 clinical trial of a vasopressin V1a receptor antagonist shows improved adaptive behaviors in men with autism spectrum disorder. Sci Transl Med.

[71] Delaglio F., Grzesiek S., Vuister GeertenW., Zhu G., Pfeifer J., Bax A.d. (1995). NMRPipe: A multidimensional spectral processing system based on UNIX pipes. J Biomol NMR.

[72] Lee W., Tonelli M., Markley J.L. (2015). NMRFAM-SPARKY: enhanced software for biomolecular NMR spectroscopy. Bioinformatics.

[73] Syed Ibrahim B., Pattabhi V. (2005). Trypsin inhibition by a peptide hormone: crystal structure of trypsin-vasopressin complex. J Mol Biol.

[74] Krieger E., Vriend G. (2014). YASARA view—molecular graphics for all devices—from smartphones to workstations. Bioinformatics.

[75] Krieger E., Koraimann G., Vriend G. (2002). Increasing the precision of comparative models with YASARA NOVA—a self-parameterizing force field. Proteins Struct Funct Bioinforma.

[76] Doshi U., Hamelberg D. (2009). Reoptimization of the AMBER force field parameters for peptide bond (omega) torsions using accelerated molecular dynamics. J Phys Chem B.

[77] Essmann U. (1995). A smooth particle mesh Ewald method. J Chem Phys.

[78] Petersen H.G. (1995). Accuracy and efficiency of the particle mesh Ewald method. J Chem Phys.

