# Supplementary material for: Conformational selection of vasopressin upon V1a receptor binding
Source: Comput Struct Biotechnol J. 2021 Oct 18;19:5826–33. doi: 10.1016/j.csbj.2021.10.024 (PMC8567363; doi:10.1016/j.csbj.2021.10.024)
Supplement: Supplementary data 1 [file mmc1.docx]

**Conformational selection of vasopressin upon V_1a_ receptor binding**

*Kateryna Che^1^, Markus Muttenthaler^1,2^, Dennis Kurzbach^1,^**

*^1^University Vienna, Faculty of Chemistry, Institute of Biological Chemistry, Währinger Str. 38, A-1090, Vienna, Austria*

*^2^ The University of Queensland, Institute for Molecular Bioscience, 306 Carmody Rd, 4072 St Lucia, Brisbane, Queensland, Australia*

**corresponding author. E-Mail:* [*dennis.kurzbach@univie.ac.at*](mailto:dennis.kurzbach@univie.ac.at)

**Supporting Information**

**
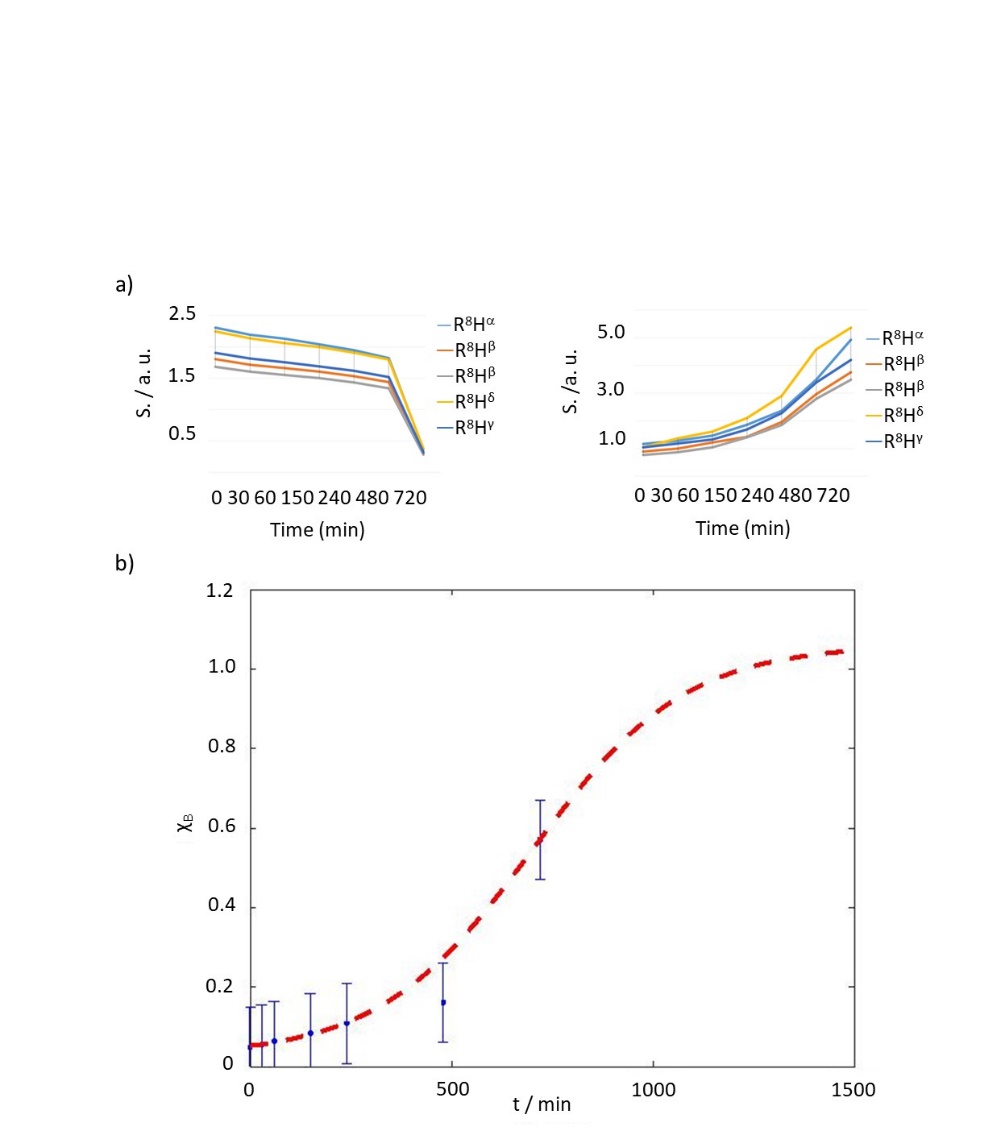
**

**Figure S1. Intensities changes of signals belonging to residue R^8^**. a) Intensities changes of residue R^8^ resonances with heating time for the trans and cis forms of proline residue 7, respectively. The sigmoidal development indicates increasing population of the *trans* state upon annealing.

**
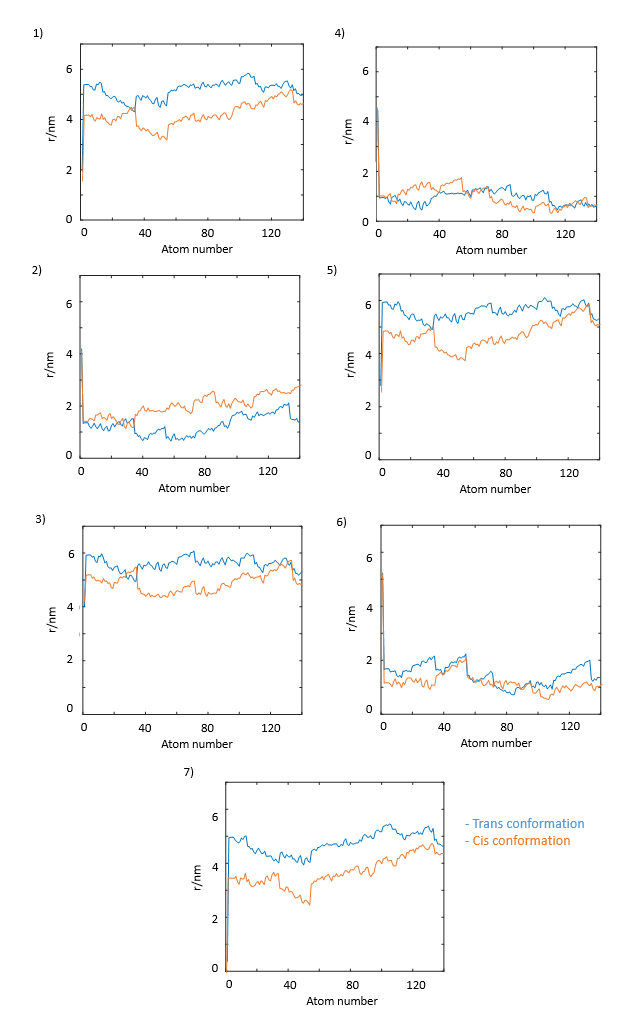
Figure S2**. **Distance differences in cis and trans conformations.**  Distances r between the VP atoms and the center of gravity of V_1a_R TMHs 1 to 7. The tail interacts similarly in both cases with H4, while differences in interaction with H6 can be inferred from different intermolecular distances.


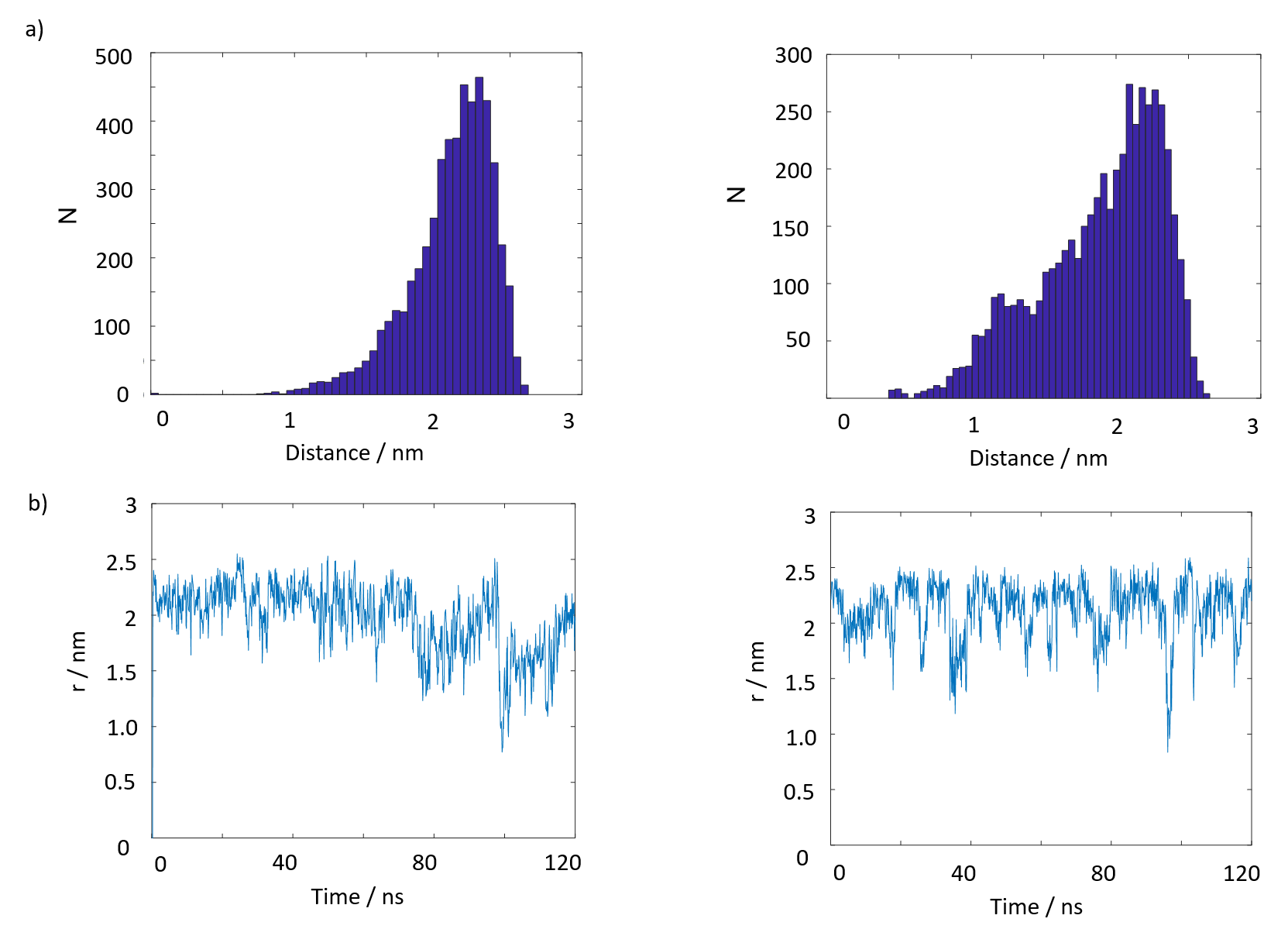


**Figure S3. Distance distributions between Arg^8^ and Tyr^2^** **in MD simulations of VP with Pro^7^ in cis state**. a) Distributions of Arg^8^(H^δ^)-Tyr^2^(H^δ^) distances r found in MD simulations of VP in the Pro^7^-cis state (replica 1 – left, replica 2 – right). While the former displayed a bimodal distribution with two distinct maxima, the latter showed only a single maximum. b) Trajectories of Arg^8^(H^δ^)-Tyr^2^(H^δ^) distances r underlying the distributions in panel a.


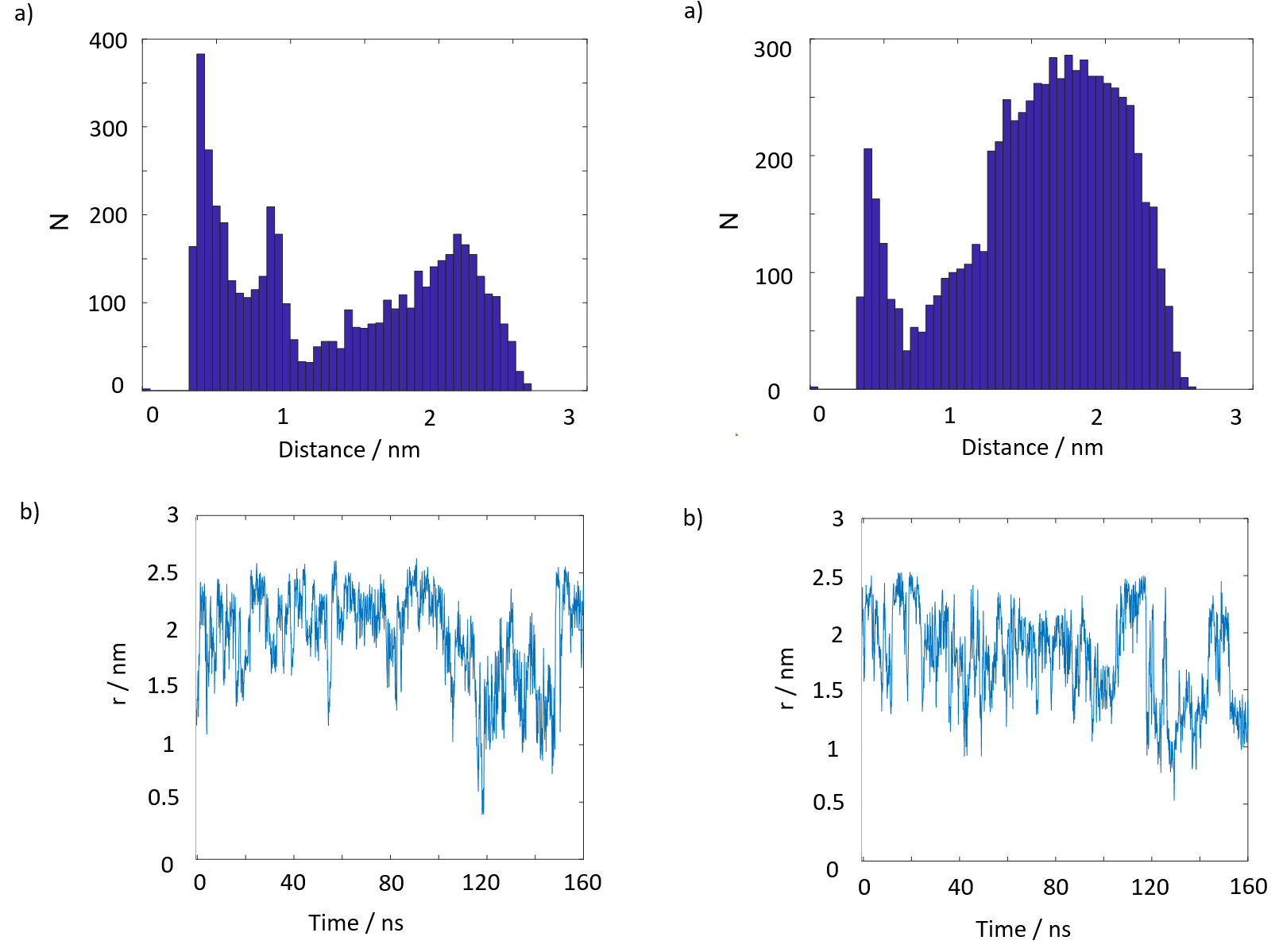


**Figure S4. Distance distributions between Arg^8^ and Tyr^2^** **in MD simulations of VP with Pro^7^ in trans state.** a) Distributions of Arg^8^(H^δ^)-Tyr^2^(H^δ^) distances r found in MD simulations of VP in the Pro^7^-trans state (replica 1 – left, replica 2 – right). While the former displayed a bimodal distribution with two distinct maxima, the latter showed only a single maximum. b) Trajectories of Arg^8^(H^δ^)-Tyr^2^(H^δ^) distances r underlying the distributions in panel a.

**Table S1**. VINA-derived *K_D_* values in units of nM, found in the three different runs for sub ensembles E_T_, C_T_ and E_C_.

**TE1 TC1 CE1 TE2 TC2 CE2 TE3 TC3 CE3**

| 38.061 | 57.165 | 50.624 | 17.217 | 45.364 | 33.991 | 38.971 | 71.673 | 64.552 |
| --- | --- | --- | --- | --- | --- | --- | --- | --- |
| 38.061 | 57.165 | 50.624 | 17.217 | 45.364 | 33.991 | 38.971 | 71.673 | 64.552 |
| 40.995 | 58.236 | 62.409 | 27.111 | 47.319 | 48.043 | 64.552 | 72.28 | 67.22 |
| 40.995 | 58.236 | 62.409 | 27.111 | 47.319 | 48.043 | 64.552 | 72.28 | 67.22 |
| 43.489 | 59.228 | 70.236 | 36.859 | 49.525 | 50.029 | 86.88 | 74.51 | 70.236 |
| 43.489 | 59.228 | 70.236 | 36.859 | 49.525 | 50.029 | 86.88 | 74.51 | 70.236 |
| 49.861 | 66.881 | 81.758 | 38.839 | 50.881 | 64.443 | 87.764 | 82.312 | 87.616 |
| 49.861 | 66.881 | 81.758 | 38.839 | 50.881 | 64.443 | 87.764 | 82.312 | 87.616 |
| 50.283 | 67.334 | 87.321 | 41.203 | 51.485 | 74.384 | 89.711 | 84.994 | 92.948 |
| 50.283 | 67.334 | 87.321 | 41.203 | 51.485 | 74.384 | 89.711 | 84.994 | 92.948 |
| 52.097 | 68.596 | 89.258 | 47.72 | 56.398 | 74.888 | 92.791 | 97.117 | 100.45 |
| 52.097 | 68.596 | 89.258 | 47.72 | 56.398 | 74.888 | 92.791 | 97.117 | 100.45 |
| 52.628 | 73.016 | 91.238 | 47.8 | 57.262 | 79.579 | 106.74 | 97.61 | 104.43 |
| 52.628 | 73.016 | 91.238 | 47.8 | 57.262 | 79.579 | 106.74 | 97.61 | 104.43 |
| 54.068 | 73.884 | 97.281 | 48.369 | 57.65 | 83.149 | 108.2 | 104.78 | 107.47 |
| 54.068 | 73.884 | 97.281 | 48.369 | 57.65 | 83.149 | 108.2 | 104.78 | 107.47 |
| 54.251 | 79.714 | 101.65 | 53.523 | 59.629 | 87.468 | 112.67 | 105.49 | 108.74 |
| 54.251 | 79.714 | 101.65 | 53.523 | 59.629 | 87.468 | 112.67 | 105.49 | 108.74 |
| 59.328 | 84.708 | 102.16 | 54.434 | 61.676 | 88.209 | 118.12 | 111.34 | 111.91 |
| 59.328 | 84.708 | 102.16 | 54.434 | 61.676 | 88.209 | 118.12 | 111.34 | 111.91 |
| 59.528 | 84.851 | 105.49 | 54.618 | 62.938 | 89.862 | 127.87 | 113.24 | 112.48 |
| 59.528 | 84.851 | 105.49 | 54.618 | 62.938 | 89.862 | 127.87 | 113.24 | 112.48 |
| 59.932 | 85.859 | 107.47 | 54.618 | 68.944 | 96.627 | 131.15 | 121.97 | 115.56 |
| 59.932 | 85.859 | 107.47 | 54.618 | 68.944 | 96.627 | 131.15 | 121.97 | 115.56 |
| 60.439 | 86.587 | 108.2 | 58.532 | 70.117 | 96.953 | 150.11 | 125.94 | 118.32 |
| 60.439 | 86.587 | 108.2 | 58.532 | 70.117 | 96.953 | 150.11 | 125.94 | 118.32 |
| 61.572 | 88.957 | 109.48 | 59.932 | 71.431 | 97.94 | 152.67 | 126.8 | 126.8 |
| 61.572 | 88.957 | 109.48 | 59.932 | 71.431 | 97.94 | 152.67 | 126.8 | 126.8 |
| 61.989 | 91.701 | 111.34 | 61.78 | 71.794 | 100.96 | 153.18 | 127.23 | 127.44 |
| 61.989 | 91.701 | 111.34 | 61.78 | 71.794 | 100.96 | 153.18 | 127.23 | 127.44 |
| 62.409 | 96.79 | 114.01 | 62.832 | 73.139 | 102.85 | 159.51 | 133.61 | 133.83 |
| 62.409 | 96.79 | 114.01 | 64.661 | 73.139 | 102.85 | 160.05 | 133.61 | 133.83 |
| 63.471 | 96.79 | 114.78 | 67.561 | 73.387 | 108.74 | 160.05 | 134.97 | 135.88 |
| 63.471 | 96.79 | 114.78 | 70.831 | 73.387 | 108.74 | 162.5 | 134.97 | 135.88 |
| 64.334 | 104.25 | 115.36 | 70.831 | 74.51 | 114.2 | 162.5 | 135.65 | 138.43 |
| 64.334 | 104.25 | 115.36 | 72.77 | 74.51 | 114.2 | 164.99 | 135.65 | 138.43 |
| 66.207 | 104.78 | 116.73 | 73.884 | 76.549 | 122.18 | 164.99 | 136.34 | 138.43 |
| 66.207 | 104.78 | 116.73 | 73.884 | 76.549 | 122.18 | 173.85 | 136.34 | 138.43 |
| 66.431 | 106.03 | 117.92 | 75.395 | 77.984 | 122.18 | 173.85 | 136.57 | 139.37 |
| 66.431 | 111.72 | 117.92 | 75.395 | 77.984 | 122.18 | 178.01 | 136.57 | 139.37 |
| 66.655 | 111.72 | 121.56 | 76.679 | 81.345 | 125.52 | 178.01 | 136.8 | 141.98 |
| 66.655 | 113.24 | 121.56 | 76.679 | 83.29 | 125.52 | 181.35 | 136.8 | 141.98 |
| 66.768 | 113.24 | 124.68 | 78.645 | 83.854 | 125.52 | 182.58 | 137.5 | 142.22 |
| 66.768 | 116.93 | 124.68 | 78.645 | 83.854 | 126.16 | 187.89 | 137.5 | 142.22 |
| 68.134 | 116.93 | 127.01 | 83.571 | 84.708 | 126.16 | 187.89 | 140.07 | 146.36 |
| 68.134 | 118.32 | 127.01 | 83.571 | 84.708 | 132.04 | 191.41 | 140.07 | 146.36 |
| 71.552 | 118.32 | 127.87 | 87.468 | 87.616 | 132.04 | 191.41 | 141.02 | 146.85 |
| 71.552 | 120.54 | 127.87 | 87.468 | 87.616 | 134.97 | 191.41 | 141.02 | 148.35 |
| 73.387 | 120.54 | 128.96 | 89.711 | 90.777 | 134.97 | 191.41 | 141.5 | 148.35 |
| 73.387 | 123.42 | 128.96 | 89.711 | 90.777 | 136.8 | 199.66 | 141.5 | 148.85 |
| 74.259 | 123.42 | 129.61 | 89.862 | 92.791 | 136.8 | 199.66 | 141.74 | 148.85 |
| 74.259 | 124.05 | 129.61 | 89.862 | 97.775 | 137.96 | 203.4 | 141.74 | 149.61 |
| 75.268 | 124.05 | 129.61 | 90.318 | 97.775 | 137.96 | 203.4 | 141.74 | 149.61 |
| 75.268 | 127.01 | 129.61 | 90.318 | 100.28 | 138.2 | 207.21 | 141.74 | 150.62 |
| 76.291 | 127.01 | 130.49 | 91.856 | 100.28 | 138.2 | 207.21 | 143.91 | 150.62 |
| 76.291 | 130.27 | 130.49 | 95.653 | 100.96 | 141.5 | 213.97 | 143.91 | 152.92 |
| 76.549 | 130.27 | 132.26 | 95.653 | 100.96 | 144.88 | 220.94 | 146.36 | 154.74 |
| 76.549 | 132.71 | 132.26 | 97.94 | 101.3 | 146.61 | 220.94 | 146.36 | 154.74 |
| 80.119 | 132.71 | 132.49 | 97.94 | 101.3 | 146.61 | 225.46 | 153.44 | 155.79 |
| 80.119 | 132.93 | 132.49 | 98.437 | 101.65 | 146.61 | 225.46 | 153.44 | 155.79 |
| 83.854 | 137.73 | 133.16 | 98.437 | 101.65 | 146.61 | 226.6 | 155 | 156.05 |
| 83.854 | 137.73 | 133.16 | 100.96 | 101.65 | 146.85 | 226.6 | 155 | 156.05 |
| 84.28 | 137.96 | 133.61 | 100.96 | 101.65 | 146.85 | 226.99 | 155.79 | 159.51 |
| 84.28 | 137.96 | 133.61 | 101.65 | 103.55 | 146.85 | 226.99 | 156.05 | 159.51 |
| 84.851 | 138.9 | 134.06 | 101.65 | 103.55 | 146.85 | 228.14 | 156.05 | 159.78 |
| 84.851 | 138.9 | 134.06 | 104.43 | 103.9 | 148.6 | 228.14 | 165.27 | 159.78 |
| 86.733 | 142.7 | 134.51 | 104.43 | 103.9 | 148.6 | 235.18 | 165.27 | 163.33 |
| 86.733 | 142.7 | 134.51 | 104.78 | 104.78 | 149.61 | 235.18 | 166.67 | 163.33 |
| 86.733 | 143.42 | 134.97 | 104.78 | 104.78 | 149.61 | 235.57 | 166.67 | 164.16 |
| 86.733 | 143.42 | 134.97 | 105.49 | 109.67 | 149.86 | 235.57 | 180.43 | 164.16 |
| 86.733 | 143.91 | 136.11 | 105.49 | 111.91 | 149.86 | 236.37 | 180.43 | 165.27 |
| 86.733 | 143.91 | 136.11 | 107.11 | 111.91 | 152.41 | 236.37 | 185.06 | 165.27 |
| 88.061 | 144.64 | 137.03 | 107.11 | 113.43 | 152.41 | 237.97 | 185.06 | 169.22 |
| 88.061 | 144.64 | 137.03 | 107.65 | 113.43 | 154.22 | 237.97 | 186.94 | 169.22 |
| 89.258 | 145.87 | 138.2 | 107.65 | 114.39 | 154.22 | 239.58 | 186.94 | 169.22 |
| 89.258 | 145.87 | 138.2 | 108.93 | 114.39 | 155.26 | 239.58 | 199.66 | 169.22 |
| 89.408 | 146.11 | 139.13 | 108.93 | 117.33 | 155.26 | 239.99 | 199.66 | 169.51 |
| 89.408 | 146.11 | 139.13 | 109.3 | 117.33 | 157.38 | 239.99 | 201.69 | 170.66 |
| 92.948 | 146.61 | 141.5 | 109.3 | 118.52 | 160.05 | 239.99 | 201.69 | 170.66 |
| 92.948 | 146.61 | 141.5 | 109.85 | 119.52 | 160.05 | 239.99 | 202.72 | 171.52 |
| 93.105 | 160.32 | 147.6 | 110.78 | 119.52 | 160.05 | 240.39 | 204.09 | 171.52 |
| 93.105 | 160.32 | 147.6 | 110.78 | 120.13 | 160.6 | 240.39 | 204.43 | 172.98 |
| 93.262 | 160.87 | 147.85 | 111.72 | 120.13 | 160.6 | 241.21 | 204.43 | 172.98 |
| 93.262 | 160.87 | 149.86 | 111.72 | 123.84 | 160.87 | 242.43 | 204.78 | 173.27 |
| 94.37 | 161.41 | 150.11 | 113.05 | 123.84 | 160.87 | 244.9 | 204.78 | 174.44 |
| 94.37 | 161.41 | 150.11 | 113.05 | 124.26 | 163.05 | 244.9 | 204.78 | 174.44 |
| 94.37 | 163.88 | 153.96 | 113.05 | 124.26 | 163.6 | 250.75 | 206.17 | 180.13 |
| 94.37 | 163.88 | 154.74 | 114.01 | 125.52 | 163.6 | 251.18 | 206.17 | 180.13 |
| 95.653 | 164.71 | 154.74 | 114.01 | 125.52 | 165.27 | 251.18 | 206.86 | 181.65 |
| 95.653 | 164.71 | 155 | 114.59 | 128.3 | 165.27 | 252.45 | 206.86 | 181.65 |
| 96.464 | 167.52 | 159.51 | 114.59 | 128.3 | 166.95 | 252.45 | 208.27 | 186.63 |
| 96.464 | 167.52 | 159.51 | 115.75 | 129.61 | 166.95 | 252.88 | 208.27 | 186.63 |
| 97.446 | 172.68 | 163.88 | 115.75 | 129.61 | 170.37 | 254.16 | 210.03 | 188.21 |
| 97.446 | 172.68 | 163.88 | 116.54 | 130.05 | 170.37 | 261.12 | 210.03 | 188.21 |
| 98.271 | 177.41 | 165.27 | 117.52 | 130.05 | 170.37 | 262 | 211.1 | 190.77 |
| 98.271 | 177.41 | 166.11 | 117.52 | 130.27 | 170.37 | 262.45 | 211.1 | 190.77 |
| 99.439 | 179.22 | 166.11 | 117.52 | 130.27 | 172.39 | 263.33 | 212.17 | 190.77 |
| 99.439 | 179.22 | 169.79 | 118.32 | 130.27 | 172.39 | 263.33 | 214.33 | 190.77 |
| 102.85 | 179.52 | 169.79 | 118.32 | 130.27 | 173.56 | 269.17 | 214.33 | 190.77 |
| 102.85 | 179.52 | 170.66 | 118.92 | 131.37 | 173.56 | 269.17 | 218.34 | 190.77 |
| 105.67 | 180.13 | 170.66 | 118.92 | 131.37 | 174.15 | 272.37 | 220.19 | 195.99 |
| 105.67 | 180.13 | 170.94 | 118.92 | 131.82 | 179.52 | 273.29 | 220.19 | 195.99 |
| 105.67 | 181.65 | 170.94 | 118.92 | 131.82 | 179.52 | 279.36 | 220.57 | 197.98 |
| 105.67 | 181.65 | 171.23 | 120.33 | 132.26 | 179.52 | 279.36 | 220.57 | 198.32 |
| 107.11 | 181.96 | 171.23 | 123.84 | 132.26 | 179.52 | 285.07 | 225.08 | 198.32 |
| 107.83 | 181.96 | 171.23 | 124.26 | 135.43 | 179.82 | 285.07 | 225.46 | 198.99 |
| 107.83 | 182.58 | 171.23 | 124.26 | 138.66 | 179.82 | 287.97 | 225.46 | 198.99 |
| 107.83 | 182.58 | 171.23 | 127.23 | 144.64 | 179.82 | 287.97 | 228.52 | 199.66 |
| 108.2 | 185.37 | 171.52 | 127.44 | 144.64 | 180.13 | 288.95 | 228.91 | 199.66 |
| 108.2 | 185.37 | 171.52 | 127.87 | 145.37 | 180.13 | 290.42 | 228.91 | 201.69 |
| 110.41 | 185.68 | 174.44 | 127.87 | 145.37 | 185.68 | 290.42 | 229.3 | 201.69 |
| 110.41 | 185.68 | 174.44 | 129.39 | 147.1 | 185.68 | 298.87 | 229.3 | 202.03 |
| 110.6 | 186 | 175.92 | 129.39 | 147.1 | 186.31 | 308.09 | 231.63 | 202.03 |
| 110.78 | 186 | 175.92 | 129.83 | 149.61 | 186.31 | 308.09 | 231.63 | 202.72 |
| 110.78 | 186.63 | 181.35 | 129.83 | 149.61 | 187.89 | 312.8 | 232.81 | 202.72 |
| 111.16 | 191.09 | 181.65 | 132.49 | 150.87 | 187.89 | 313.33 | 232.81 | 205.13 |
| 111.16 | 191.09 | 181.65 | 132.49 | 150.87 | 189.8 | 316.52 | 235.18 | 205.82 |
| 112.48 | 191.09 | 182.88 | 132.49 | 151.13 | 189.8 | 316.52 | 235.18 | 205.82 |
| 112.48 | 191.09 | 189.8 | 132.71 | 152.92 | 193.03 | 318.13 | 237.17 | 214.69 |
| 113.43 | 191.41 | 189.8 | 132.71 | 156.85 | 193.03 | 318.13 | 237.17 | 214.69 |
| 113.43 | 191.41 | 189.8 | 134.06 | 156.85 | 194.34 | 321.37 | 239.18 | 214.69 |
| 113.43 | 194.01 | 190.77 | 134.06 | 158.17 | 194.34 | 321.37 | 239.58 | 214.69 |
| 113.43 | 194.01 | 190.77 | 134.29 | 158.17 | 194.34 | 324.09 | 239.58 | 218.34 |
| 115.75 | 198.32 | 191.73 | 135.43 | 158.44 | 196.98 | 326.28 | 240.8 | 218.34 |
| 115.75 | 198.32 | 191.73 | 139.6 | 160.87 | 196.98 | 326.28 | 241.21 | 219.82 |
| 115.95 | 198.99 | 193.36 | 140.31 | 160.87 | 201.69 | 327.94 | 245.31 | 219.82 |
| 115.95 | 198.99 | 193.36 | 140.31 | 162.5 | 201.69 | 327.94 | 245.31 | 220.57 |
| 116.34 | 202.03 | 194.34 | 140.79 | 162.5 | 203.06 | 335.22 | 247.39 | 220.57 |
| 116.34 | 202.03 | 194.34 | 140.79 | 162.5 | 203.06 | 336.92 | 247.39 | 225.08 |
| 117.72 | 203.06 | 197.31 | 141.5 | 162.5 | 204.43 | 343.23 | 264.22 | 228.91 |
| 117.72 | 203.06 | 197.31 | 142.46 | 165.55 | 204.43 | 343.23 | 264.22 | 229.68 |
| 118.32 | 207.91 | 197.65 | 145.62 | 165.55 | 205.47 | 345.56 | 265.12 | 229.68 |
| 118.32 | 207.91 | 197.65 | 145.62 | 166.11 | 206.17 | 346.73 | 265.12 | 230.07 |
| 119.32 | 207.91 | 198.32 | 146.61 | 166.11 | 206.17 | 351.44 | 269.63 | 230.07 |
| 120.94 | 207.91 | 198.32 | 146.61 | 166.11 | 207.21 | 351.44 | 269.63 | 230.46 |
| 120.94 | 209.68 | 199.32 | 146.85 | 166.39 | 207.21 | 351.44 | 271.46 | 230.46 |
| 122.18 | 209.68 | 199.32 | 148.35 | 166.39 | 208.27 | 352.63 | 271.46 | 232.02 |
| 124.26 | 210.74 | 199.66 | 150.87 | 168.08 | 208.27 | 353.22 | 272.37 | 232.02 |
| 124.47 | 210.74 | 200.34 | 150.87 | 168.08 | 208.27 | 353.82 | 273.76 | 233.99 |
| 124.47 | 211.45 | 200.34 | 151.38 | 168.37 | 208.62 | 356.82 | 274.68 | 234.38 |
| 125.31 | 213.24 | 200.67 | 151.38 | 168.37 | 208.62 | 356.82 | 274.68 | 234.38 |
| 125.94 | 213.24 | 200.67 | 151.64 | 169.51 | 210.03 | 361.67 | 275.61 | 234.78 |
| 127.44 | 218.34 | 200.67 | 151.64 | 169.51 | 210.03 | 369.69 | 275.61 | 234.78 |
| 127.44 | 218.34 | 201.35 | 152.15 | 169.51 | 210.03 | 372.2 | 277.01 | 234.78 |
| 127.66 | 224.7 | 201.69 | 152.15 | 169.79 | 210.03 | 375.35 | 282.2 | 239.58 |
| 127.66 | 224.7 | 202.72 | 152.15 | 169.79 | 215.05 | 375.35 | 283.63 | 239.58 |
| 127.87 | 225.46 | 202.72 | 152.15 | 169.79 | 215.05 | 375.35 | 283.63 | 245.31 |
| 127.87 | 225.46 | 204.09 | 152.41 | 169.79 | 219.08 | 375.98 | 283.63 | 245.31 |
| 129.83 | 225.46 | 206.51 | 153.7 | 172.1 | 220.94 | 375.98 | 283.63 | 246.14 |
| 129.83 | 226.6 | 208.27 | 153.96 | 172.1 | 220.94 | 377.89 | 283.63 | 246.56 |
| 130.93 | 226.6 | 208.27 | 155.79 | 172.39 | 226.22 | 377.89 | 290.42 | 246.56 |
| 130.93 | 227.75 | 210.74 | 156.58 | 172.39 | 226.22 | 378.53 | 294.36 | 246.97 |
| 131.82 | 228.14 | 210.74 | 156.58 | 173.85 | 229.68 | 379.81 | 294.36 | 246.97 |
| 131.82 | 228.14 | 210.74 | 157.11 | 175.03 | 234.38 | 381.74 | 295.86 | 247.39 |
| 132.04 | 232.02 | 210.74 | 157.11 | 175.92 | 234.38 | 381.74 | 296.36 | 248.65 |
| 132.04 | 232.02 | 211.1 | 157.91 | 175.92 | 235.18 | 384.97 | 296.36 | 248.65 |
| 134.51 | 232.81 | 211.1 | 160.6 | 175.92 | 235.18 | 385.62 | 297.86 | 252.45 |
| 134.51 | 232.81 | 211.81 | 160.87 | 175.92 | 237.17 | 385.62 | 297.86 | 252.45 |
| 134.51 | 232.81 | 211.81 | 160.87 | 179.82 | 237.17 | 386.28 | 300.89 | 253.73 |
| 134.97 | 237.57 | 212.17 | 161.14 | 179.82 | 237.57 | 390.87 | 302.42 | 254.16 |
| 134.97 | 237.57 | 212.17 | 161.14 | 180.74 | 238.37 | 391.53 | 308.09 | 254.16 |
| 136.8 | 238.37 | 213.61 | 164.16 | 180.74 | 240.39 | 392.85 | 308.09 | 255.45 |
| 141.26 | 238.37 | 214.33 | 164.16 | 180.74 | 240.39 | 392.85 | 314.39 | 255.45 |
| 141.26 | 244.49 | 214.33 | 164.44 | 180.74 | 240.8 | 393.51 | 314.39 | 255.88 |
| 141.74 | 249.07 | 215.78 | 164.44 | 180.74 | 244.07 | 394.18 | 317.06 | 255.88 |
| 141.74 | 249.07 | 217.98 | 164.99 | 181.96 | 244.07 | 394.85 | 318.13 | 259.36 |
| 141.98 | 251.18 | 218.71 | 164.99 | 183.5 | 246.56 | 396.18 | 318.67 | 259.36 |
| 142.7 | 251.18 | 223.19 | 165.55 | 183.5 | 246.56 | 398.19 | 321.37 | 262.89 |
| 142.7 | 253.73 | 223.19 | 166.11 | 186.31 | 246.97 | 398.86 | 321.91 | 262.89 |
| 142.7 | 253.73 | 223.56 | 166.11 | 186.31 | 247.81 | 402.24 | 323.54 | 266.46 |
| 143.18 | 258.49 | 226.6 | 166.67 | 188.84 | 247.81 | 403.6 | 326.28 | 266.46 |
| 143.18 | 258.49 | 226.6 | 166.67 | 188.84 | 251.6 | 404.97 | 327.94 | 268.27 |
| 144.15 | 261.12 | 230.46 | 166.95 | 189.16 | 252.03 | 404.97 | 329.61 | 269.17 |
| 145.87 | 261.12 | 230.46 | 167.8 | 189.48 | 252.45 | 408.4 | 329.61 | 269.17 |
| 146.36 | 261.56 | 231.24 | 168.08 | 189.48 | 255.88 | 416.05 | 332.4 | 271.46 |
| 146.61 | 261.56 | 231.24 | 168.37 | 190.12 | 261.12 | 426.72 | 332.4 | 271.46 |
| 146.61 | 265.12 | 232.41 | 168.94 | 190.44 | 261.12 | 426.72 | 333.52 | 273.29 |
| 146.61 | 265.12 | 232.81 | 169.22 | 190.77 | 264.67 | 432.52 | 333.52 | 273.29 |
| 146.61 | 267.36 | 233.59 | 169.79 | 190.77 | 264.67 | 432.52 | 334.65 | 273.76 |
| 150.87 | 267.36 | 233.59 | 169.79 | 192.71 | 264.67 | 433.99 | 334.65 | 273.76 |
| 150.87 | 271.46 | 234.38 | 169.79 | 193.36 | 265.56 | 439.14 | 338.06 | 275.15 |
| 151.13 | 271.46 | 235.97 | 170.08 | 193.36 | 265.56 | 439.14 | 338.06 | 275.15 |
| 151.13 | 276.54 | 235.97 | 170.08 | 195.66 | 266.91 | 439.14 | 339.2 | 278.42 |
| 151.38 | 276.54 | 236.77 | 170.08 | 195.66 | 269.63 | 439.14 | 339.2 | 278.42 |
| 154.48 | 277.48 | 238.37 | 170.37 | 196.98 | 269.63 | 439.14 | 344.39 | 278.89 |
| 154.48 | 277.48 | 241.21 | 170.37 | 198.32 | 270.54 | 440.63 | 345.56 | 283.63 |
| 157.64 | 279.83 | 241.21 | 170.37 | 198.65 | 270.54 | 446.62 | 349.66 | 283.63 |
| 160.05 | 279.83 | 242.43 | 170.37 | 198.65 | 274.22 | 447.37 | 349.66 | 286.52 |
| 160.05 | 286.52 | 242.43 | 173.27 | 199.32 | 274.22 | 448.13 | 350.25 | 286.52 |
| 160.87 | 286.52 | 247.81 | 173.27 | 199.32 | 274.68 | 448.13 | 352.03 | 287 |
| 160.87 | 287.49 | 247.81 | 177.11 | 199.66 | 275.61 | 448.89 | 353.82 | 287.49 |
| 161.68 | 289.93 | 247.81 | 177.11 | 199.66 | 276.08 | 448.89 | 353.82 | 287.49 |
| 161.68 | 289.93 | 247.81 | 177.11 | 200.67 | 276.08 | 464.3 | 354.42 | 290.91 |
| 163.05 | 289.93 | 250.33 | 178.01 | 200.67 | 277.01 | 465.87 | 355.02 | 291.4 |
| 163.88 | 289.93 | 250.33 | 178.31 | 201.01 | 277.48 | 465.87 | 355.02 | 291.4 |
| 163.88 | 290.42 | 250.75 | 178.61 | 201.01 | 277.48 | 467.44 | 358.63 | 291.89 |
| 163.88 | 290.42 | 250.75 | 178.61 | 201.35 | 278.42 | 476.2 | 361.06 | 294.36 |
| 163.88 | 290.91 | 251.18 | 179.52 | 201.35 | 278.42 | 478.62 | 362.28 | 294.36 |
| 166.39 | 290.91 | 251.18 | 179.52 | 205.82 | 280.3 | 480.24 | 366.58 | 296.36 |
| 166.39 | 293.37 | 251.6 | 179.82 | 206.17 | 280.3 | 481.86 | 366.58 | 297.86 |
| 166.95 | 296.36 | 251.6 | 180.13 | 207.56 | 280.78 | 481.86 | 368.45 | 297.86 |
| 167.52 | 300.89 | 254.16 | 182.58 | 207.56 | 281.72 | 481.86 | 368.45 | 298.36 |
| 168.65 | 300.89 | 254.16 | 182.58 | 207.91 | 282.68 | 482.68 | 371.57 | 298.36 |
| 168.65 | 307.05 | 254.16 | 183.5 | 207.91 | 284.11 | 482.68 | 371.57 | 299.88 |
| 169.79 | 307.05 | 254.16 | 183.5 | 211.1 | 284.11 | 482.68 | 373.45 | 303.44 |
| 169.79 | 308.09 | 255.45 | 184.75 | 211.1 | 285.07 | 487.59 | 382.38 | 303.44 |
| 170.94 | 308.09 | 255.45 | 185.06 | 211.1 | 285.07 | 487.59 | 382.38 | 304.47 |
| 170.94 | 309.13 | 255.88 | 185.68 | 211.81 | 285.07 | 489.24 | 384.33 | 307.05 |
| 170.94 | 309.65 | 255.88 | 185.68 | 212.53 | 285.55 | 499.25 | 384.33 | 308.09 |
| 170.94 | 309.65 | 256.75 | 185.68 | 212.53 | 285.55 | 506.03 | 388.24 | 309.65 |
| 170.94 | 310.17 | 256.75 | 185.68 | 214.69 | 287.97 | 512.91 | 392.85 | 309.65 |
| 170.94 | 310.17 | 258.49 | 186 | 214.69 | 288.46 | 519.89 | 400.89 | 310.17 |
| 171.23 | 312.28 | 258.49 | 189.16 | 216.51 | 288.95 | 520.76 | 402.92 | 310.17 |
| 173.85 | 312.28 | 258.93 | 189.16 | 218.71 | 288.95 | 525.18 | 402.92 | 311.22 |
| 174.15 | 312.8 | 258.93 | 190.77 | 218.71 | 291.89 | 525.18 | 407.71 | 315.45 |
| 174.15 | 315.99 | 258.93 | 192.38 | 218.71 | 293.37 | 527.84 | 413.26 | 315.45 |
| 174.15 | 315.99 | 258.93 | 192.38 | 218.71 | 293.37 | 531.42 | 414.65 | 316.52 |
| 174.74 | 315.99 | 260.68 | 192.71 | 219.08 | 293.37 | 532.32 | 415.35 | 316.52 |
| 174.74 | 318.67 | 260.68 | 194.34 | 219.08 | 295.36 | 535.92 | 415.35 | 319.2 |
| 174.74 | 318.67 | 260.68 | 195.33 | 220.57 | 295.36 | 539.55 | 418.17 | 319.2 |
| 174.74 | 318.67 | 260.68 | 198.32 | 220.57 | 295.36 | 539.55 | 418.17 | 319.2 |
| 174.74 | 318.67 | 261.56 | 198.32 | 222.06 | 295.86 | 567.58 | 418.17 | 319.2 |
| 174.74 | 320.28 | 261.56 | 199.66 | 222.44 | 295.86 | 567.58 | 419.58 | 319.2 |
| 175.62 | 320.28 | 261.56 | 201.01 | 222.44 | 298.36 | 568.53 | 421.71 | 319.2 |
| 175.62 | 321.37 | 263.78 | 201.01 | 224.7 | 302.93 | 570.46 | 421.71 | 319.74 |
| 175.62 | 321.37 | 265.12 | 205.82 | 224.7 | 302.93 | 575.29 | 423.85 | 321.91 |
| 175.92 | 321.91 | 265.12 | 205.82 | 225.08 | 304.98 | 576.26 | 428.17 | 323 |
| 175.92 | 322.45 | 265.56 | 207.21 | 225.08 | 305.5 | 580.17 | 432.52 | 323 |
| 175.92 | 325.19 | 267.36 | 207.91 | 225.46 | 310.17 | 586.07 | 433.25 | 325.19 |
| 175.92 | 325.19 | 268.72 | 207.91 | 225.46 | 310.17 | 587.06 | 436.19 | 326.28 |
| 176.51 | 329.05 | 268.72 | 208.27 | 226.6 | 310.17 | 594.04 | 436.19 | 326.28 |
| 177.71 | 329.05 | 269.17 | 208.27 | 226.6 | 311.22 | 594.04 | 438.4 | 328.5 |
| 180.13 | 331.28 | 269.17 | 208.62 | 227.37 | 311.22 | 595.04 | 438.4 | 329.05 |
| 180.13 | 331.28 | 269.63 | 208.97 | 227.37 | 311.75 | 596.05 | 442.12 | 329.05 |
| 182.27 | 332.96 | 269.63 | 208.97 | 227.37 | 311.75 | 598.06 | 442.12 | 330.72 |
| 184.75 | 334.09 | 270.54 | 213.24 | 227.37 | 312.28 | 603.13 | 449.64 | 330.72 |
| 185.68 | 335.78 | 275.15 | 214.33 | 227.75 | 312.28 | 603.13 | 451.16 | 335.22 |
| 185.68 | 335.78 | 277.48 | 214.69 | 227.75 | 312.8 | 603.13 | 455.76 | 335.22 |
| 185.68 | 335.78 | 280.3 | 215.78 | 227.75 | 312.8 | 610.3 | 455.76 | 338.63 |
| 185.68 | 336.35 | 280.3 | 218.71 | 227.75 | 312.8 | 610.3 | 461.95 | 341.5 |
| 185.68 | 337.49 | 280.3 | 218.71 | 228.14 | 312.8 | 613.4 | 461.95 | 341.5 |
| 185.68 | 337.49 | 284.11 | 219.82 | 228.14 | 313.33 | 613.4 | 466.65 | 343.23 |
| 186.94 | 338.06 | 284.11 | 221.69 | 229.3 | 313.33 | 613.4 | 476.2 | 343.23 |
| 186.94 | 340.35 | 285.07 | 221.69 | 229.68 | 313.86 | 617.55 | 485.13 | 344.39 |
| 186.94 | 340.35 | 285.55 | 222.06 | 232.41 | 314.39 | 617.55 | 486.77 | 344.39 |
| 188.21 | 340.92 | 285.55 | 225.08 | 232.81 | 315.45 | 617.55 | 487.59 | 345.56 |
| 188.21 | 340.92 | 287 | 225.08 | 233.99 | 315.45 | 618.6 | 488.41 | 345.56 |
| 188.52 | 340.92 | 287.49 | 225.84 | 234.78 | 316.52 | 623.84 | 489.24 | 346.14 |
| 188.52 | 342.65 | 288.95 | 225.84 | 238.37 | 317.06 | 627.01 | 490.89 | 354.42 |
| 188.52 | 343.23 | 288.95 | 228.14 | 238.37 | 318.13 | 628.07 | 493.38 | 355.62 |
| 188.52 | 343.23 | 289.93 | 229.68 | 240.8 | 319.2 | 632.32 | 493.38 | 356.22 |
| 188.84 | 343.81 | 289.93 | 229.68 | 240.8 | 319.2 | 632.32 | 500.09 | 356.22 |
| 188.84 | 344.97 | 291.4 | 230.85 | 240.8 | 320.82 | 634.46 | 508.6 | 362.89 |
| 189.16 | 345.56 | 291.4 | 233.2 | 243.25 | 320.82 | 637.68 | 510.32 | 362.89 |
| 189.16 | 345.56 | 293.37 | 233.2 | 244.9 | 320.82 | 637.68 | 515.52 | 363.5 |
| 190.44 | 345.56 | 293.37 | 234.78 | 244.9 | 320.82 | 643.08 | 515.52 | 365.97 |
| 190.44 | 345.56 | 293.37 | 236.37 | 246.97 | 323.54 | 648.53 | 521.64 | 367.82 |
| 191.41 | 346.73 | 293.87 | 237.97 | 246.97 | 323.54 | 648.53 | 521.64 | 372.82 |
| 191.41 | 346.73 | 293.87 | 238.78 | 246.97 | 324.09 | 656.24 | 522.52 | 372.82 |
| 191.41 | 347.9 | 297.86 | 239.58 | 248.65 | 324.09 | 656.24 | 522.52 | 373.45 |
| 191.41 | 347.9 | 300.39 | 239.58 | 248.65 | 327.39 | 658.46 | 532.32 | 373.45 |
| 191.41 | 348.49 | 302.42 | 240.39 | 249.07 | 329.61 | 665.16 | 532.32 | 374.09 |
| 191.41 | 348.49 | 303.44 | 240.39 | 250.75 | 329.61 | 665.16 | 535.92 | 374.09 |
| 191.73 | 352.03 | 303.44 | 241.61 | 250.75 | 330.16 | 673.07 | 536.83 | 374.09 |
| 193.36 | 352.63 | 304.98 | 242.43 | 251.18 | 332.96 | 674.2 | 542.29 | 374.72 |
| 193.36 | 356.82 | 306.01 | 243.25 | 254.16 | 332.96 | 678.77 | 547.81 | 374.72 |
| 194.67 | 356.82 | 306.01 | 243.25 | 254.16 | 334.09 | 681.07 | 551.52 | 374.72 |
| 195.33 | 356.82 | 308.09 | 243.66 | 254.16 | 335.22 | 695 | 551.52 | 375.35 |
| 195.33 | 358.02 | 312.28 | 245.31 | 254.59 | 337.49 | 698.53 | 555.26 | 381.1 |
| 195.66 | 361.06 | 312.28 | 246.56 | 254.59 | 337.49 | 699.71 | 556.2 | 381.1 |
| 195.99 | 362.89 | 312.8 | 247.39 | 258.49 | 342.07 | 704.45 | 558.08 | 383.03 |
| 195.99 | 362.89 | 313.33 | 247.39 | 258.49 | 342.07 | 705.64 | 558.08 | 383.68 |
| 197.31 | 363.5 | 316.52 | 247.81 | 258.93 | 343.23 | 708.02 | 558.08 | 383.68 |
| 197.31 | 363.5 | 318.13 | 247.81 | 258.93 | 343.23 | 708.02 | 559.02 | 386.28 |
| 197.98 | 365.97 | 320.28 | 247.81 | 259.36 | 343.81 | 715.23 | 559.02 | 386.28 |
| 200.34 | 365.97 | 320.28 | 248.65 | 259.36 | 344.39 | 720.08 | 561.86 | 386.93 |
| 200.34 | 365.97 | 321.37 | 248.65 | 260.68 | 344.39 | 724.95 | 567.58 | 386.93 |
| 200.67 | 366.58 | 324.09 | 249.49 | 260.68 | 346.73 | 724.95 | 567.58 | 389.55 |
| 200.67 | 366.58 | 324.64 | 250.33 | 260.68 | 346.73 | 727.41 | 575.29 | 391.53 |
| 200.67 | 367.82 | 324.64 | 250.33 | 262.45 | 349.07 | 727.41 | 580.17 | 391.53 |
| 200.67 | 367.82 | 328.5 | 252.45 | 264.67 | 353.22 | 733.57 | 582.13 | 392.19 |
| 202.72 | 369.07 | 331.28 | 253.31 | 266.01 | 353.22 | 736.05 | 582.13 | 392.19 |
| 202.72 | 369.07 | 331.28 | 253.31 | 266.01 | 353.22 | 738.54 | 584.1 | 392.85 |
| 203.06 | 373.45 | 332.4 | 253.31 | 266.01 | 353.22 | 739.79 | 585.08 | 393.51 |
| 203.4 | 373.45 | 332.96 | 254.16 | 266.01 | 354.42 | 746.06 | 585.08 | 394.18 |
| 204.09 | 373.45 | 332.96 | 258.93 | 266.46 | 354.42 | 767.77 | 587.06 | 395.51 |
| 204.09 | 374.09 | 336.92 | 259.36 | 266.46 | 356.22 | 772.97 | 588.05 | 398.19 |
| 204.43 | 374.72 | 336.92 | 260.24 | 267.81 | 359.23 | 775.59 | 588.05 | 402.24 |
| 206.86 | 374.72 | 338.06 | 261.12 | 267.81 | 359.84 | 776.9 | 598.06 | 404.29 |
| 206.86 | 375.35 | 338.63 | 261.12 | 268.27 | 359.84 | 790.12 | 598.06 | 404.97 |
| 208.62 | 375.35 | 340.35 | 262.45 | 268.27 | 361.67 | 794.13 | 600.09 | 404.97 |
| 209.68 | 375.98 | 344.97 | 263.78 | 272.37 | 361.67 | 800.86 | 601.1 | 407.03 |
| 209.68 | 375.98 | 346.14 | 265.56 | 272.37 | 364.12 | 817.25 | 603.13 | 407.03 |
| 210.03 | 377.26 | 349.07 | 265.56 | 274.22 | 364.12 | 829.76 | 610.3 | 411.86 |
| 210.38 | 377.26 | 349.07 | 270.08 | 274.22 | 364.73 | 841.04 | 610.3 | 411.86 |
| 210.38 | 380.45 | 349.66 | 270.54 | 276.54 | 366.58 | 841.04 | 612.36 | 415.35 |
| 212.17 | 384.33 | 349.66 | 271 | 276.54 | 366.58 | 862.6 | 612.36 | 417.46 |
| 212.17 | 386.93 | 350.85 | 275.61 | 277.95 | 370.32 | 868.45 | 613.4 | 418.87 |
| 212.89 | 386.93 | 350.85 | 276.08 | 278.42 | 370.32 | 869.91 | 613.4 | 418.87 |
| 212.89 | 390.87 | 352.03 | 277.01 | 278.42 | 372.2 | 871.38 | 619.64 | 418.87 |
| 212.89 | 390.87 | 352.03 | 277.01 | 278.42 | 372.82 | 871.38 | 625.95 | 418.87 |
| 214.33 | 390.87 | 352.03 | 277.48 | 278.89 | 373.45 | 875.81 | 634.46 | 419.58 |
| 214.33 | 396.18 | 356.82 | 277.95 | 278.89 | 373.45 | 883.23 | 639.83 | 419.58 |
| 217.61 | 396.18 | 356.82 | 281.72 | 283.15 | 374.09 | 883.23 | 642 | 420.29 |
| 217.98 | 398.86 | 358.63 | 288.95 | 283.15 | 374.09 | 887.71 | 654.03 | 420.29 |
| 217.98 | 398.86 | 358.63 | 288.95 | 283.15 | 375.98 | 892.22 | 657.35 | 421 |
| 217.98 | 398.86 | 361.06 | 290.91 | 284.11 | 377.89 | 905.87 | 657.35 | 422.42 |
| 217.98 | 409.78 | 362.28 | 290.91 | 284.59 | 377.89 | 905.87 | 657.35 | 422.42 |
| 218.71 | 409.78 | 363.5 | 291.4 | 287 | 378.53 | 908.94 | 659.57 | 426 |
| 218.71 | 413.95 | 364.73 | 291.89 | 287 | 379.17 | 910.47 | 665.16 | 426 |
| 218.71 | 415.35 | 365.35 | 294.36 | 287.97 | 380.45 | 913.55 | 666.29 | 427.44 |
| 218.71 | 415.35 | 365.35 | 295.86 | 287.97 | 381.74 | 918.19 | 666.29 | 427.44 |
| 219.08 | 418.87 | 365.97 | 295.86 | 289.44 | 382.38 | 922.85 | 667.41 | 429.61 |
| 219.08 | 418.87 | 365.97 | 297.36 | 289.44 | 382.38 | 936.98 | 669.67 | 429.61 |
| 219.82 | 419.58 | 367.2 | 297.36 | 289.44 | 383.68 | 952.92 | 675.34 | 432.52 |
| 220.19 | 420.29 | 367.2 | 297.36 | 290.42 | 383.68 | 964.25 | 675.34 | 433.25 |
| 220.94 | 421 | 367.2 | 297.36 | 290.42 | 385.62 | 965.88 | 678.77 | 433.25 |
| 220.94 | 425.28 | 367.82 | 299.88 | 290.42 | 385.62 | 977.36 | 691.49 | 434.72 |
| 220.94 | 427.44 | 369.07 | 299.88 | 290.91 | 385.62 | 1000.7 | 693.83 | 436.19 |
| 221.69 | 427.44 | 369.07 | 299.88 | 290.91 | 387.58 | 1014.3 | 693.83 | 436.19 |
| 221.69 | 427.44 | 370.32 | 299.88 | 292.38 | 391.53 | 1016 | 702.07 | 439.14 |
| 222.44 | 428.17 | 372.82 | 299.88 | 293.87 | 392.19 | 1019.5 | 708.02 | 440.63 |
| 222.44 | 428.17 | 377.89 | 299.88 | 293.87 | 398.86 | 1021.2 | 723.73 | 441.37 |
| 224.7 | 431.07 | 379.17 | 300.89 | 294.86 | 399.54 | 1033.3 | 723.73 | 441.37 |
| 225.84 | 431.79 | 379.81 | 300.89 | 296.36 | 399.54 | 1038.6 | 723.73 | 442.87 |
| 226.6 | 433.25 | 379.81 | 301.4 | 297.36 | 399.54 | 1042.1 | 723.73 | 445.11 |
| 226.6 | 439.14 | 380.45 | 302.93 | 298.36 | 400.21 | 1059.8 | 726.18 | 446.62 |
| 226.6 | 439.14 | 386.28 | 303.96 | 298.36 | 400.21 | 1063.4 | 727.41 | 447.37 |
| 226.6 | 440.63 | 386.28 | 303.96 | 301.4 | 403.6 | 1063.4 | 731.1 | 450.4 |
| 227.37 | 440.63 | 388.24 | 305.5 | 301.91 | 403.6 | 1065.2 | 736.05 | 451.93 |
| 228.14 | 447.37 | 388.24 | 305.5 | 302.42 | 403.6 | 1130 | 751.11 | 451.93 |
| 228.14 | 447.37 | 389.55 | 306.01 | 302.42 | 403.6 | 1133.9 | 760.04 | 454.22 |
| 228.52 | 448.13 | 389.55 | 306.53 | 304.47 | 403.6 | 1135.8 | 775.59 | 454.22 |
| 230.85 | 448.13 | 390.87 | 308.09 | 304.98 | 403.6 | 1161 | 775.59 | 454.22 |
| 230.85 | 448.89 | 390.87 | 309.13 | 306.01 | 404.97 | 1180.7 | 776.9 | 454.22 |
| 230.85 | 448.89 | 391.53 | 309.13 | 306.01 | 404.97 | 1188.7 | 778.21 | 456.53 |
| 230.85 | 454.99 | 391.53 | 309.13 | 306.53 | 404.97 | 1206.9 | 778.21 | 459.62 |
| 231.24 | 456.53 | 393.51 | 309.13 | 306.53 | 404.97 | 1209 | 783.48 | 459.62 |
| 231.24 | 458.84 | 393.51 | 310.17 | 308.09 | 407.71 | 1223.3 | 783.48 | 465.87 |
| 231.63 | 460.4 | 395.51 | 310.17 | 310.17 | 408.4 | 1244.2 | 787.46 | 468.23 |
| 233.59 | 460.4 | 399.54 | 310.7 | 313.33 | 409.09 | 1250.5 | 791.46 | 471.4 |
| 233.59 | 467.44 | 400.89 | 311.22 | 313.33 | 411.17 | 1269.6 | 791.46 | 474.6 |
| 233.59 | 467.44 | 400.89 | 311.22 | 313.33 | 411.86 | 1276.1 | 795.47 | 477.01 |
| 233.59 | 477.01 | 401.57 | 311.22 | 315.45 | 411.86 | 1278.2 | 795.47 | 477.81 |
| 233.59 | 477.01 | 401.57 | 311.75 | 315.99 | 411.86 | 1278.2 | 795.47 | 477.81 |
| 233.59 | 477.81 | 402.92 | 312.8 | 315.99 | 411.86 | 1284.7 | 795.47 | 479.43 |
| 233.59 | 477.81 | 402.92 | 313.86 | 315.99 | 412.56 | 1313.2 | 799.51 | 479.43 |
| 233.99 | 481.86 | 402.92 | 313.86 | 315.99 | 413.26 | 1317.6 | 800.86 | 484.31 |
| 234.78 | 484.31 | 402.92 | 314.39 | 315.99 | 413.26 | 1335.5 | 802.22 | 484.31 |
| 239.18 | 487.59 | 405.65 | 315.99 | 317.06 | 413.95 | 1346.9 | 804.93 | 485.13 |
| 239.18 | 487.59 | 405.65 | 323 | 317.59 | 414.65 | 1372.1 | 804.93 | 485.13 |
| 240.39 | 489.24 | 408.4 | 323 | 317.59 | 414.65 | 1395.5 | 804.93 | 485.95 |
| 240.39 | 489.24 | 409.09 | 324.09 | 317.59 | 416.05 | 1426.4 | 809.01 | 485.95 |
| 240.39 | 490.06 | 411.17 | 324.09 | 317.59 | 417.46 | 1445.8 | 813.12 | 486.77 |
| 240.8 | 494.22 | 415.35 | 325.19 | 319.2 | 417.46 | 1450.7 | 813.12 | 486.77 |
| 241.21 | 494.22 | 417.46 | 325.73 | 319.2 | 423.85 | 1480.4 | 814.49 | 490.06 |
| 242.84 | 494.22 | 417.46 | 325.73 | 320.28 | 424.57 | 1498 | 839.62 | 494.22 |
| 242.84 | 500.94 | 422.42 | 325.73 | 320.28 | 425.28 | 1533.8 | 842.46 | 494.22 |
| 242.84 | 500.94 | 423.85 | 325.73 | 321.91 | 425.28 | 1541.6 | 849.6 | 494.22 |
| 243.66 | 500.94 | 423.85 | 325.73 | 324.64 | 425.28 | 1573.1 | 851.03 | 495.89 |
| 243.66 | 503.48 | 425.28 | 326.84 | 324.64 | 425.28 | 1573.1 | 858.25 | 495.89 |
| 244.07 | 503.48 | 426 | 327.39 | 326.28 | 427.44 | 1597.2 | 858.25 | 496.73 |
| 244.49 | 504.33 | 426 | 327.94 | 328.5 | 427.44 | 1638.2 | 859.7 | 497.56 |
| 247.81 | 504.33 | 429.61 | 327.94 | 328.5 | 428.17 | 1643.7 | 866.98 | 501.78 |
| 247.81 | 504.33 | 429.61 | 327.94 | 329.05 | 428.17 | 1643.7 | 872.85 | 504.33 |
| 247.81 | 504.33 | 430.34 | 328.5 | 329.05 | 429.61 | 1649.2 | 872.85 | 504.33 |
| 247.81 | 506.89 | 431.79 | 328.5 | 329.05 | 429.61 | 1685.8 | 872.85 | 505.18 |
| 249.07 | 517.26 | 433.25 | 329.05 | 330.72 | 430.34 | 1685.8 | 874.33 | 509.46 |
| 249.49 | 517.26 | 433.25 | 329.05 | 331.28 | 430.34 | 1705.9 | 887.71 | 510.32 |
| 249.49 | 517.26 | 435.45 | 329.61 | 331.84 | 432.52 | 1749.6 | 892.22 | 511.18 |
| 250.33 | 520.76 | 435.45 | 329.61 | 331.84 | 432.52 | 1779.4 | 912.01 | 516.39 |
| 250.75 | 525.18 | 436.93 | 331.84 | 332.4 | 433.25 | 1809.7 | 915.09 | 516.39 |
| 250.75 | 530.52 | 437.66 | 331.84 | 332.4 | 433.25 | 1831.2 | 916.64 | 517.26 |
| 250.75 | 532.32 | 438.4 | 334.09 | 332.4 | 433.25 | 1846.7 | 916.64 | 518.13 |
| 251.18 | 535.02 | 442.12 | 335.22 | 332.4 | 433.25 | 1881.3 | 921.29 | 525.18 |
| 251.18 | 545.97 | 442.87 | 336.92 | 335.22 | 434.72 | 1919.8 | 924.41 | 526.06 |
| 251.18 | 545.97 | 443.61 | 337.49 | 337.49 | 434.72 | 1972.4 | 933.82 | 527.84 |
| 251.18 | 545.97 | 443.61 | 337.49 | 338.06 | 435.45 | 1985.7 | 938.56 | 527.84 |
| 251.18 | 547.81 | 444.36 | 342.07 | 338.06 | 435.45 | 1999.2 | 941.73 | 529.63 |
| 251.18 | 553.39 | 445.87 | 342.65 | 338.63 | 435.45 | 2040.1 | 943.32 | 529.63 |
| 252.03 | 553.39 | 447.37 | 342.65 | 338.63 | 436.93 | 2043.5 | 946.51 | 529.63 |
| 252.03 | 554.32 | 447.37 | 342.65 | 338.63 | 440.63 | 2053.9 | 946.51 | 530.52 |
| 252.88 | 567.58 | 448.13 | 346.14 | 340.92 | 445.87 | 2053.9 | 951.32 | 530.52 |
| 253.31 | 567.58 | 449.64 | 348.49 | 340.92 | 445.87 | 2149.7 | 954.53 | 531.42 |
| 253.31 | 575.29 | 449.64 | 353.82 | 341.5 | 448.89 | 2182.6 | 961 | 534.12 |
| 253.73 | 577.24 | 449.64 | 356.22 | 342.65 | 448.89 | 2234.7 | 965.88 | 535.92 |
| 253.73 | 577.24 | 450.4 | 356.82 | 346.14 | 450.4 | 2323.2 | 965.88 | 545.04 |
| 253.73 | 579.19 | 452.69 | 359.23 | 347.31 | 451.93 | 2362.7 | 969.14 | 545.97 |
| 254.59 | 579.19 | 454.99 | 359.23 | 347.31 | 451.93 | 2523.5 | 975.71 | 545.97 |
| 254.59 | 579.19 | 458.07 | 359.23 | 347.31 | 454.22 | 2532 | 979.01 | 545.97 |
| 255.88 | 582.13 | 458.84 | 359.23 | 347.31 | 456.53 | 2536.3 | 979.01 | 553.39 |
| 255.88 | 588.05 | 459.62 | 359.84 | 349.07 | 456.53 | 2579.5 | 980.66 | 555.26 |
| 257.18 | 589.05 | 459.62 | 361.06 | 350.85 | 457.3 | 2579.5 | 980.66 | 555.26 |
| 257.62 | 591.04 | 459.62 | 361.67 | 350.85 | 457.3 | 2583.8 | 980.66 | 558.08 |
| 258.05 | 591.04 | 461.17 | 364.73 | 351.44 | 457.3 | 2614.6 | 987.31 | 558.08 |
| 258.93 | 593.04 | 462.73 | 364.73 | 352.03 | 458.07 | 2632.3 | 999.04 | 558.08 |
| 259.36 | 593.04 | 463.51 | 364.73 | 352.03 | 459.62 | 2632.3 | 999.04 | 558.08 |
| 260.24 | 594.04 | 463.51 | 369.07 | 352.63 | 459.62 | 2690.7 | 1005.8 | 560.91 |
| 260.68 | 596.05 | 469.02 | 369.07 | 352.63 | 459.62 | 2690.7 | 1005.8 | 560.91 |
| 262.45 | 597.06 | 469.02 | 369.07 | 353.82 | 465.08 | 2987.5 | 1005.8 | 563.76 |


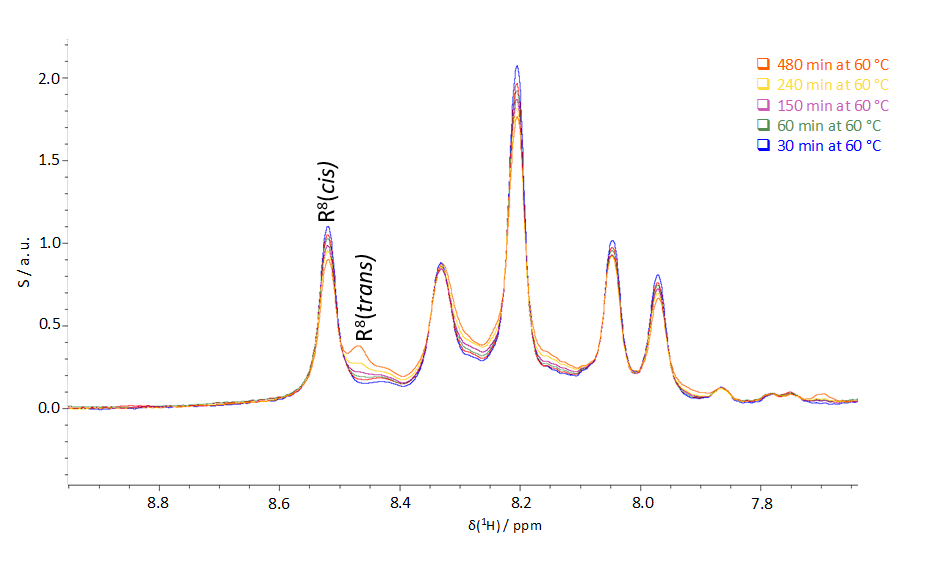


**Figure S5.** The annealing process monitored by ^1^H-^1^H TOCSY of VP. By heating the sample, the contribution of the Pro^7^ *trans* form increases as can be monitored from the two respective Arg^8^ signals (indicated in the figure). The spectra were all recorded at 25°C (after cooling down subsequent to heating the sample). Note that signals stemming from the *trans* conformation are broader than those stemming from the *cis* contributions.

**Figure S6.** 1D ^1^H spectra of VP at 25°C and 60°C. The amide peaks drop significantly in intensity in the high-temperature case, since accelerated proton exchange broadens the signals. Similarly, data analysis is impeded due to the strong signal overlap.


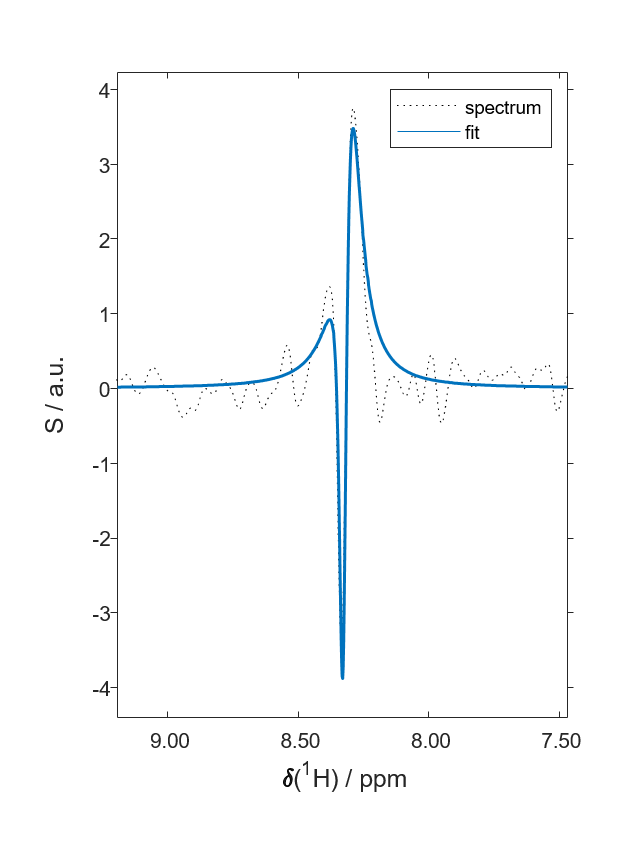


**Figure S7.** 1D projection of the NOESY shown in Fig. 3 of the main text focusing on R^8^(H^α^) - G^9^(H^N^) cross peak. The blue line indicates a fit if the spectrum to two Lorentzian functions. As can be seen, the Pro^7^-*cis* form leads to a share negative signal and the Pro^7^-*trans* form to a broadened positive signal. The broadened signal stretches even to the region down-field of the sharp negative signal.


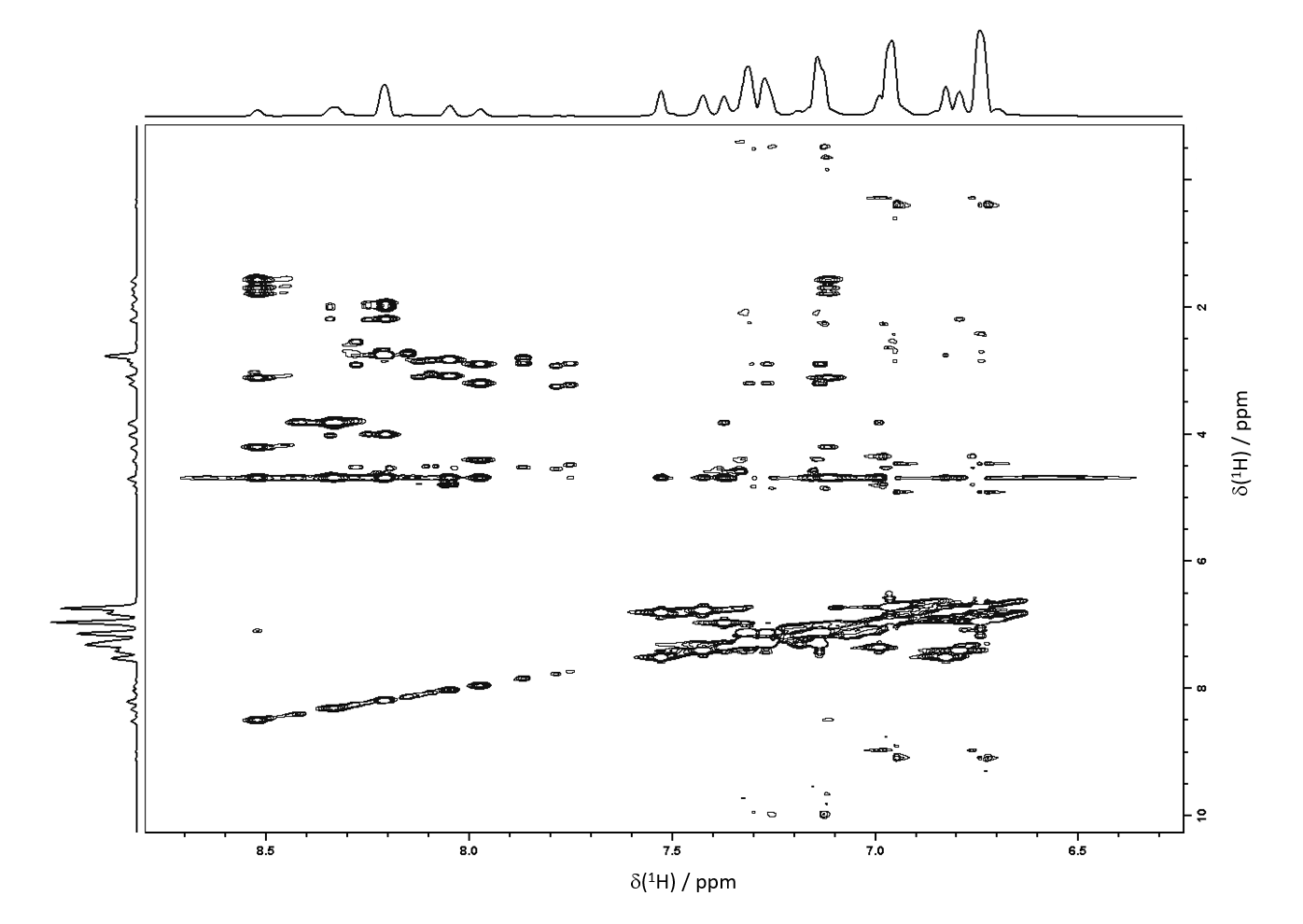


**Figure S8.** ^1^H-^1^H TOCSY of VP (the same as in the main text) with 1D projections.
